# Structural characterization and colour of Mg_x_Cu_3-x_V_2_O_8_ (0 ≤ x ≤ 3) and Mg_y_Cu_2-y_V_2_O_7_ (0 ≤ y ≤ 2) compositions

**DOI:** 10.1186/s40064-015-0908-8

**Published:** 2015-04-03

**Authors:** M A Tena, Santiago García-Granda

**Affiliations:** Inorganic Chemistry Area, Inorganic and Organic Chemistry Department, Jaume I University, P.O. Box 224, Castellón, Spain; Physical Chemistry Area, Physical and Analytical Chemistry Department, Oviedo University-CINN, Oviedo, Spain

**Keywords:** Orthovanadate, Divanadate, Solid solutions, Structure, Colour

## Abstract

In this study, Mg_x_Cu_3-x_V_2_O_8_ (0 ≤ x ≤ 3) and Mg_y_Cu_2-y_V_2_O_7_ (0 ≤ y ≤ 2) compositions were synthesized by the chemical coprecipitation method and characterized by X-ray diffraction, UV–vis-NIR spectroscopy and CIE L* a* b* parameters measurements. Melting points of compounds Cu_3_V_2_O_8_ and Cu_2_V_2_O_7_ are 780°C and 790°C, respectively. The addition of small amounts of Mg (II), Mg_x_Cu_3-x_V_2_O_8_ (x < 1.0) and Mg_y_Cu_2-y_V_2_O_7_ (y < 0.5) fused compositions, was not sufficient to stabilize structures at 800°C. For the Mg_2_CuV_2_O_8_ (x = 2.0) composition fired at 800°C, Mg (II) incorporated into the monoclinic Cu_3_V_2_O_8_ structure stabilizes this crystalline phase. At 1000°C, orthorhombic Mg_3_V_2_O_8_ structure from this composition was obtained. Solid solutions with orthorhombic symmetry were detected from the prepared compositions fired at 1000°C when 1.0 ≤ x ≤ 3.0. The difference of coloration of Cu, Mg vanadates might be explained by the presence of a strong charge transfer band in visible spectra.

**Electronic supplementary material:**

The online version of this article (doi:10.1186/s40064-015-0908-8) contains supplementary material, which is available to authorized users.

## Background

The preparation of materials with applications on ceramic pigments industry by the conventional ceramic method presents some drawbacks. Thus, the necessary high temperatures and long soaking times give rise to loss of volatile reagents and consequently to deviation of the stoichiometric conditions of the initial systems. Some improvements were obtained with alternative synthesis methods (García et al. 2001, Tena et al. 2003). For example, no desirable brown materials were obtained from oxide mixtures in V_x_Ti_1-x_O_2_ (x < 0.10) rutile solid solutions with potential usefulness as gray ceramic pigments but only bluish gray colorations were obtained from gels (Tena et al. 2003). However, the ceramic pigments industry tends towards cheap and simple processing. The synthesis from mixtures of solid starting materials with addition of halides as flux agents is habitual (Sorlí et al. 2004). Alternative synthetic methods are used exceptionally. The choice of precursors materials is a possibility to obtain desired final industrial product. The bright colours of vanadates could be considered from ceramic industry to development ceramic pigments but their melting points are low. These melting points can be modified by the formation of solid solutions (West 1984). Solid solutions are very common in crystalline materials. A solid solution is basically a crystalline phase that can have variable composition.

Cu_3_V_2_O_8_ and Mg_3_V_2_O_8_ compounds melt incongruently at 780°C and 1212°C respectively (Fleury 1966, Clark and Morley 1976). Mg_2_V_2_O_7_ melts incongruently at 1132°C (Clark and Morley 1976) and Cu_2_V_2_O_7_ melts at 790°C (Fleury 1966). Considering these melting points, Mg (II) orthovanadate and Mg (II) divanadate structures can be more suitable for ceramic industry than Cu (II) orthovanadate and Cu (II) divanadate structures.

Colour is often, but not always, associated with transition metal ions. In molecular chemistry, colour can arise from two possible common causes. The d-d electronic transitions within transition metal ions give rise to many of the familiar colours of transition metal compounds, e. g. the various shades of blue and green associated with different copper (II) complexes. Charge transfer effects in which an electron is transferred between an anion and a cation are often responsible for intense colours as in, for example, permanganate (purple) and chromates (yellow). In solids, there is an additional source of colour; it involves the transition of electrons between energy bands. Colour may be measured in a quantitative way by spectroscopic techniques. At higher frequencies than the infrared, electronic transitions associated with d-level splitting, impurity ions, crystal defects, etc., are possible. Many of these occur in the visible region and are responsible for colour.

Most of divalent metal orthovanadates of small ionic radius have orthorhombic symmetry. Copper orthovanadate presents triclinic symmetry at atmospheric pressure (Coing-Boyat 1982). A monoclinic form of this compound prepared under pressure at high temperature (4 GPa, 1173K) was described (Shannon and Calvo 1972). The structure of Cu_3_V_2_O_8_ compound with monoclinic symmetry and space group P12_1_/c1 is similar to the orthorhombic structure of Mg_3_V_2_O_8_ compound with space group Cmca (Krishnamachari and Calvo 1971). Most of divalent metal divanadates, M_2_V_2_O_7_, are polymorphic. Copper divanadate, Cu_2_V_2_O_7_, presents orthorhombic (Calvo and Faggiani 1975), monoclinic (Mercurio-Lavaud and Bernard Frit 1973) and triclinic crystalline forms and Mg_2_V_2_O_7_ presents monoclinic and triclinic symmetries (Gopal and Calvo 1974). Information about these crystal structures is included in an Additional file 1.

In this study, structural characterization of Mg_x_Cu_3-x_V_2_O_8_ (0 ≤ x ≤ 3) and Mg_y_Cu_2-y_V_2_O_7_ (0 ≤ y ≤ 2) compositions was made to investigate the possible formation of solid solutions with orthovanadate or divanadate structure. Coloration of these materials was also measured and related with structure in prepared compositions. A partial substitution of Cu (II) by Mg (II) ions might increase the melting point of materials with orthovanadates and divanadates structures. These potential solid solutions could be applied in ceramic industry.

## Methods

Mg_x_Cu_3-x_V_2_O_8_ (0 ≤ x ≤ 3) and Mg_y_Cu_2-y_V_2_O_7_ (0 ≤ y ≤ 2) compositions were synthesized by the chemical coprecipitation method. The starting materials were Cu(NO_3_)_2_ · 2.5H_2_O (Sigma-Aldrich, 98%), MgCl_2_.6H_2_O (Panreac) of reagent grade chemical quality, and NH_4_VO_3_ (Sigma-Aldrich, 99%). The stoichiometric amount of NH_4_VO_3_, Cu(NO_3_)_2_ · 2.5H_2_O and MgCl_2_ · 6H_2_O was added on 200 mL of water with vigorous stirring at room temperature. These starting materials were added in solid state and the concentration of the various cations is different in each prepared composition. After that, a solution of ammonium hydroxide was added dropwise until pH = 8. The obtained precipitates were dried by an infrared lamp and dry samples were fired at 300°C for 12 hours, 600°C for 12 hours, 800°C for 1 hour and 1000°C for 1 hour.

The resulting materials were examined by X-ray diffraction with CuKα radiation to study the development of the crystalline phases at different temperatures. A structure profile refinement was carried out by the Rietveld method (Fullprof.2 k computer program) (Rietveld 1969, Rodriguez-Carvajal 2012, Chapon and Rodriguez-Carvajal 2008). Unit cell parameters and interatomic distances (M-O and V-O) in divanadates and orthovanadates structures were obtained from Mg_x_Cu_3-x_V_2_O_8_ (0 ≤ x ≤ 3) and Mg_y_Cu_2-y_V_2_O_7_ (0 ≤ y ≤ 2) fired compositions to investigate the possibility of formation of solid solution in these synthetic conditions. The diffraction patterns were collected running from 5 to 110 ^o^2θ, using monochromatic CuK_α_ radiation, a step size of 0.02 ^o^2θ and a sampling time of 10 s. The initial structural information was obtained of the Inorganic Crystal Structure Database (Inorganic Crystal Structure Database ICSD 2013). Table 1 includes the reference ICSD to every structure. This initial structural information also appears in the references (Coing-Boyat 1982, Shannon and Calvo 1972, Krishnamachari and Calvo 1971, Calvo and Faggiani 1975, Mercurio-Lavaud and Bernard Frit 1973, Gopal and Calvo 1974) for the main structures of this study. Dicvol program (Boultif and Loüer 2007) was used to obtain initial cell parameters in some compositions.

**Table 1:** **Structural information of Cu and Mg orthovanadates and divanadates**

**Structure**	ICSD* reference	**Crystalline system**	Standard unit cell* (Å and degrees)	**Standard space group**	**Z**
Cu_3_V_2_O_8_	27184	Triclinic	a = 5.196 (4), b = 5.355 (1), c = 6.505 (4), α = 69.22 (3), β = 88.69 (4), γ = 68.08 (3)	P −1	1
Cu_3_V_2_O_8_	27310	Monoclinic	a = 6.2493 (9), b = 7.9936 (9), c = 6.378 (1), β = 111.49 (1)	P 1 2_1_/c 1	2
MgCu_2_V_2_O_8_	404852	Monoclinic	a = 6.453 (1), b = 8.342 (2), c = 11.517 (2), β = 90.44 (2)	P 1 2_1_/c 1	4
Mg_2_CuV_2_O_8_	404851	Monoclinic	a = 6.167 (3), b = 8.172 (5), c = 6.400 (3), β = 116.22 (3)	P 1 2_1_/c 1	2
Mg_3_V_2_O_8_	156155	Orthorhombic	a = 6.0814 (7), b = 11.469 (1), c = 8.337 (1)	C m c a	4
α-Cu_2_V_2_O_7_	34756	Orthorhombic	a = 8.411 (5), b = 20.68 (1), c = 6.448 (5)	F d d 2	8
β-Cu_2_V_2_O_7_	158375	Monoclinic	a = 7.6890 (8), b = 8.0289 (9), c = 10.1065 (8), β = 110.252 (7)	C 1 2/c 1	4
γ-Cu_2_V_2_O_7_	171028	Triclinic	a = 5.087 (1), b = 5.823 (1), c = 9.402 (2), α = 99.780 (3), β = 97.253 (3), γ = 97.202 (3)	P −1	2
Cu_1.5_ Mg_0.5_V_2_O_7_	69731	Monoclinic	a = 7.660 (6), b = 8.089 (8), c = 10.117 (9), β = 110.6 (1)	C 1 2/c 1	4
Cu_1.33_ Mg_0.67_V_2_O_7_	69732	Monoclinic	a = 7.645 (4), b = 8.095 (3), c = 10.119 (3), β = 110.54 (3)	C 1 2/c 1	4
α-Mg_2_V_2_O_7_	93603	Monoclinic	a = 6.599 (1), b = 8.406 (1), c = 9.472 (2), β = 100.6085 (4)	P 1 2_1_/c 1	4
Mg_2_V_2_O_7_	2321	Triclinic	a = 4.912 (2), b = 5.414 (3), c = 10.669 (7), α = 100.36 (4), β = 102.82 (4), γ = 98.58 (4)	P −1	2

* Inorganic Crystal Structure Database (ICSD) (2013).

UV–vis-NIR spectroscopy (diffuse reflectance) allows the Cu (II) site and the V (V)-O and Cu (II)-O charge bands in samples to be studied. A Jasco V-670 spectrophotometer was used to obtain the UV–vis-NIR (ultraviolet visible near infrared) spectra in the 200 to 2500 nm range. X-Rite spectrophotometer (SP60, an illuminant D65, an observer 10°, and a reference sample of MgO) was used to obtain CIEL*a*b* colour parameters on fired samples: L* is the lightness axis (black (0) → white (100)), a* the green (−) → red (+) axis, and b* is the blue (−) → yellow (+) axis (CIE 1971).

## Results and discussion

Tables 2 and 3 show crystalline phase evolution with composition and temperature in Mg_x_Cu_3-x_V_2_O_8_ (0 ≤ x ≤ 3) and Mg_y_Cu_2-y_V_2_O_7_ (0 ≤ y ≤ 2) compositions. XRD patterns from Mg_x_Cu_3-x_V_2_O_8_ and MgyCu_2-y_V_2_O_7_ compositions are shown in Additional files 2 and 3.

**Table 2:** **Evolution of crystalline phases with temperature in Mg**_**x**_ **Cu**_**3-x**_ **V**_**2**_ **O**_**8**_ **(0 ≤ x ≤ 3) samples**

**x**	**T (°C)**	**Crystalline phases**
0.00	300	C2(s), C1(vw)
0.50	300	C2(s), MT(w), C1(vw)
1.00	300	CM(m), M(w), C2(vw)
1.50	300	CM(m)
2.00	300	M(m), CM(w)
2.50	300	M(m), MT(w)
3.00	300	M(m), MT(w)
0.00	600	CT(s), C1(m), C2(m)
0.50	600	CT(m), C2(w), M(vw), C1(vw)
1.00	600	CT(m), M(m), C2(w), MO(w), CM(vw)
1.50	600	CM(m)
2.00	600	CM(m), MO(w)
2.50	600	MO(m), M(vw)
3.00	600	MO(m), M(w)
1.00	800	MC(s)
1.50	800	CM(s), MC(s), C2(vw)
2.00	800	CM(s)
2.50	800	MO(s), M(vw)
3.00	800	MO(s), MT(w)
1.00	1000	MO(m), CM(w), M(vw)
1.50	1000	MO(m), CM(w), M(w)
2.00	1000	MO(m), M(w), MC(vw), CT(vw)
2.50	1000	MO(s), MT(vw)
3.00	1000	MO(s), MT(w)

Crystalline phases: CT = Cu_3_V_2_O_8_ (triclinic), CM = Cu_3_V_2_O_8_ (monoclinic), MC = MgCu_2_V_2_O_8_ (monoclinic), MO = Mg_3_V_2_O_8_ (orthorhombic), C1 = α-Cu_2_V_2_O_7_ (orthorhombic), C2 = β-Cu_2_V_2_O_7_ (monoclinic), C3 = γ-Cu_2_V_2_O_7_ (triclinic), M = α-Mg_2_V_2_O_7_ (monoclinic), MT = Mg_2_V_2_O_7_ (triclinic).

Diffraction peak intensity: s = strong, m = medium, w = weak, vw = very weak.

**Table 3:** **Evolution of crystalline phases with temperature in Mg**_**y**_ **Cu**_**2-y**_ **V**_**2**_ **O**_**7**_ **(0 ≤ y ≤ 2) samples**

**y**	**T (°C)**	**Crystalline phases**
0.00	300	C2(m), CT(m), M2(m), C1(w), CM(w), C3(vw)
0.25	300	C2(m), C3(m), M3(m)
0.50	300	C3(m), M2(m), M1(m), C2(w), M3(w), C1(vw)
0.75	300	CM(s), M2(m), C2(w), C3(w)
1.00	300	C3(w), M1(w), CM(vw)
1.25	300	CM(s), N(s), C2(vw)
1.50	300	C3(m), CM(vw), M3(w)
1.75	300	N(s), CM(m), C2(vw)
2.00	300	N(s), MT(w)
0.00	600	C1(s)
0.25	600	C2(s), C1(w)
0.50	600	C2(s), C2’(m), M3(w)
0.75	600	M3(s), C2(m), C2’(m)
1.00	600	M3(m), C2(m)
1.25	600	C2(m), M4(m), M3(w)
1.50	600	C2(m), M4(m), M1(w)
1.75	600	M1(s), C2(w), M4(w)
2.00	600	M1(s), M2(w)
0.50	800	C2(s), M4(m), M2(w)
0.75	800	C2(s), M3(m), C3(w)
1.00	800	M4(s), C2(w)
1.25	800	M4(s), C2(w), M3(w)
1.50	800	C1(s), M4(m), MT(vw)
1.75	800	MT(m), M4(m)
2.00	800	MT(m), M4(m)
1.50	1000	M1(m), MT(m), C2(w)
1.75	1000	M1(s), MT(w), C2(vw)
2.00	1000	MT(m), M1(w), MO(w)

Crystalline phases: C1 = α-Cu_2_V_2_O_7_ (orthorhombic), C2 = β-Cu_2_V_2_O_7_ (monoclinic, C2: β < 110.6^o^, C2’: β > 110.6°), C3 = γ-Cu_2_V_2_O_7_ (triclinic), CT = Cu_3_V_2_O_8_ (triclinic), CM = Cu_3_V_2_O_8_ (monoclinic), M = α-Mg_2_V_2_O_7_ (monoclinic, M1: β =100.6-102.0°, M2: β = 98.0-99.6°, M3: β = 94.5-97.0°, M4: β = 88.0-92.0°), MT = Mg_2_V_2_O_7_ (triclinic), N = α-Mn_2_V_2_O_7_ (triclinic, P Ῑ, Z =4 with all atoms in 2i sites, ICSD-81993), MO = Mg_3_V_2_O_8_ (orthorhombic).

Diffraction peak intensity: s = strong, m = medium, w = weak, vw = very weak.

In Mg_x_Cu_3-x_V_2_O_8_ (0 ≤ x < 1.0) compositions the major crystalline phase is the triclinic Cu_3_V_2_O_8_ polymorph at 600°C. Cu_3_V_2_O_8_ compound melts incongruently at 780°C. This fact can explain the melting of samples with a presence higher than 50% in Cu_3_V_2_O_8_ triclinic crystalline phase (small amount of Mg in compositions). This crystalline phase with triclinic structure is not detected in samples fired at 800 and 1000°C.

**Figure 1:**
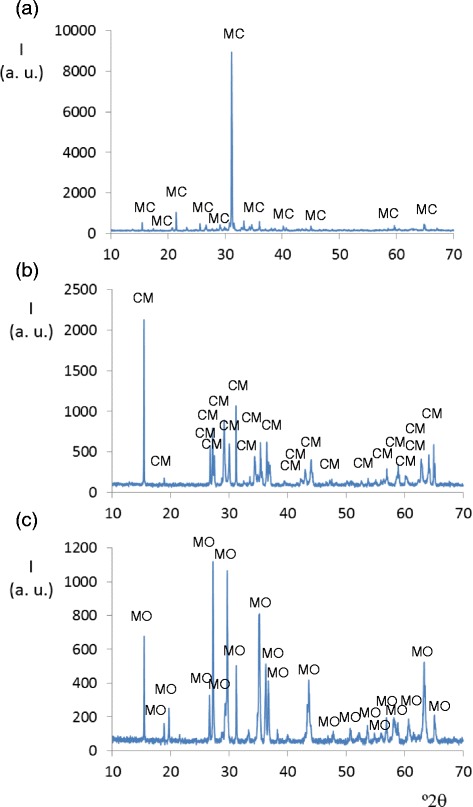
**Diffractograms from Mg**_**x**_ **Cu**_**3-x**_ **V**_**2**_ **O**_**8**_ **compositions at 800°C.** Diffraction maxima from the major crystalline phase in : **(a)** x = 1.0, MgCu_2_V_2_O_8_; **(b)** x = 2.0, Mg_2_CuV_2_O_8_ and **(c)** x = 2.5, Mg_2.5_Cu_0.5_V_2_O_8_ compositions. Crystalline phases: MC (MgCu_2_V_2_O_8_, monoclinic), CM (Cu_3_V_2_O_8_, monoclinic) and MO (Mg_3_V_2_O_8_, orthorhombic).

A crystalline phase with Cu_3_V_2_O_8_ structure and monoclinic symmetry was obtained at 300 and 600°C in compositions with 1.0 ≤ x ≤ 2.0. This crystalline phase with monoclinic Cu_3_V_2_O_8_ structure is also present when 1.5 ≤ x ≤ 2.0 at 800°C. The crystalline phase with the structure of MgCu_2_V_2_O_8_ compound (β = 90.44 (2) and Z = 4) is detected when 1.0 ≤ x ≤ 2.0 at 800°C but the crystalline phase with the ordered metal distributions in Mg_2_CuV_2_O_8_ compound (β = 116.22 (3), Z = 2, Cu1 in 2a sites and Mg1 in 4e sites, ICSD-404851) is not detected in conditions of this study. In the prepared Mg_2_CuV_2_O_8_ composition (Mg_x_Cu_3-x_V_2_O_8_ with x =2.0), crystalline phase with Cu_3_V_2_O_8_ structure and monoclinic symmetry (Z = 2 and M1 (M1 = Mg, Cu) in 2b sites and M2 (M2 = Mg, Cu) in 4e sites) is the only crystalline phase detected at 800°C. Figure 1 shows the major crystalline phase detected in MgCu_2_V_2_O_8_ (x = 1.0), Mg_2_CuV_2_O_8_ (x = 2.0) and Mg_2.5_Cu_0.5_V_2_O_8_ (x = 2.5) compositions fired at 800°C.

**Figure 2:**
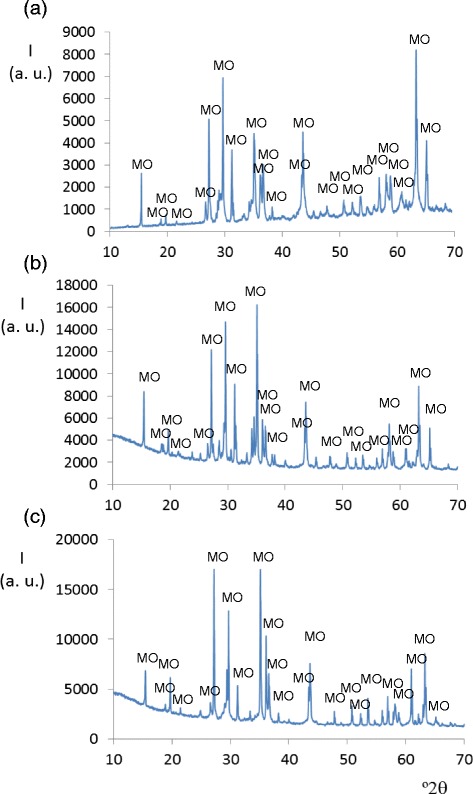
**Diffractograms from Mg**_**x**_ **Cu**_**3-x**_ **V**_**2**_ **O**_**8**_ **compositions at 1000°C.** Diffraction maxima from the major phase in: **(a)** x = 1.0, MgCu_2_V_2_O_8_; **(b)** x = 2.0, Mg_2_Cu_2_V_2_O_8_ and **(c)** x = 2.5, Mg_2.5_Cu_0.5_V_2_O_8_ compositions. MO: orthorhombic Mg_3_V_2_O_8_ crystalline phase.

Crystalline phase with orthorhombic Mg_3_V_2_O_8_ structure is obtained when 2.0 ≤ x ≤ 3.0 at 600°C and when 2.5 ≤ x ≤ 3.0 at 800°C. At 1000°C, this phase is the major crystalline phase in the unfused samples (x ≥ 1.0). Figure 2 shows the positions of diffraction lines and their intensities from Mg_x_Cu_3-x_V_2_O_8_ compositions with x = 1.0, x = 2.0 and x = 2.5 fired at 1000°C. The orthorhombic Mg_3_V_2_O_8_ crystalline phase is the major phase in them.

Cu divanadates and Mg divanadates are present together with Cu and Mg orthovanadates in some samples.

Crystalline phase with α-Cu_2_V_2_O_7_ structure (orthorhombic symmetry) is only present in Mg_y_Cu_2-y_V_2_O_7_ compositions when y < 0.5 at 600°C. These compositions melt at 800°C. Diffraction peak intensity associated with this crystalline phase is weak or very weak at 300°C.

**Figure 3:**
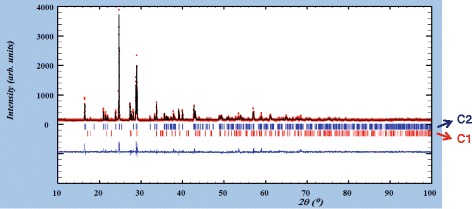
**An example of the diffraction profile refinement.** The diffraction profile refinement by Rietveld’s method from Mg_0.25_Cu_1.75_V_2_O_7_ composition fired at 600°C. Crystalline phases: α-Cu_2_V_2_O_7_ (C1) and β- Cu_2_V_2_O_7_ (C2).

Presence of crystalline phase with β-Cu_2_V_2_O_7_ structure (monoclinic symmetry) is more extended than the crystalline phase with α-Cu_2_V_2_O_7_ structure in the prepared compositions. At 300°C, crystalline phase with β-Cu_2_V_2_O_7_ structure is present when y < 0.5 with diffraction peaks of medium intensity. This crystalline phase is present when 0.25 ≤ y < 1.50 at 600°C and when 0.50 ≤ y ≤ 0.75 with diffraction peak of strong or medium intensity. Figure 3 shows graphical result of the diffraction profile refinement by Rietveld’s method from the Mg_0.25_Cu_1.75_V_2_O_7_ composition fired at 600°C.

Crystalline phase with γ-Cu_2_V_2_O_7_ structure (triclinic symmetry) is present in compositions when 0.25 ≤ y ≤ 0.50 at 300°C but its intensity of diffraction peak is weak at 600 and 800°C (Table 3).

In Mg_y_Cu_2-y_V_2_O_7_ compositions fired at 300°C, the assignation to α- Mn_2_V_2_O_7_ structure of a crystalline phase detected when y ≥ 1.25 might be explained by the existence of a polymorph of Mg_2_V_2_O_7_ compound with this structure. It was not detected any match with the existing single-crystal related in the bibliography. Synthesis of single-crystal is the most used method in synthesis of vanadates.

Crystalline phase with α-Mg_2_V_2_O_7_ structure (monoclinic symmetry) is detected in a compositional range more extended than the crystalline phase with triclinic Mg_2_V_2_O_7_ structure from Mg_y_Cu_2-y_V_2_O_7_ prepared compositions. Crystalline phase with monoclinic α-Mg_2_V_2_O_7_ structure is identified when 0.75 ≤ y ≤ 2.00 at 600°C, when 0.50 ≤ y ≤ 2.00 at 800°C, and when 1.50 ≤ y ≤ 2.00 at 1000°C.

Crystalline phase with triclinic Mg_2_V_2_O_7_ structure is observed when 1.5 ≤ y ≤ 2.00 in compositions fired at 800°C and only when y = 2.0 at 300°C.

Tables 4, 5, 6, 7, 8 and 9 show the unit cell parameters and weight fractions in crystalline phases with weight fractions higher than 8% from samples fired at 600, 800 and 1000°C. The variation of these parameters with composition confirms the formation of solid solutions.

**Table 4:** **Unit cell parameters in crystalline phases with weight fractions higher than 8% from Mg**_**x**_ **Cu**_**3-x**_ **V**_**2**_ **O**_**8**_ **(0 ≤ x ≤ 3) compositions fired at 600°C/12 h**

**x**	**Crystalline phases (weight fractions)**	**Unit cell parameters (Å and degrees)**
0.00	CT (75.02 %)	a = 5.18451(7) b = 5.34518(8) c = 6.51005(7)
		α = 69.261(1) β = 88.644(1) γ= 68.99(1)
	C1 (12.83 %)	a = 8.4016(3) b = 20.666(1) c = 6.4433(2)
		α = 90.00 β = 90.00 γ = 90.0
	C2 (12.16 %)	a = 7.6982(6) b = 8.0361(9) c = 10.112(1)
		α = 90.00 β = 110.29(1) γ = 90.00
0.50	CT (64.09 %)	a = 5.1861(2) b = 5.3448(2) c = 6.5457(2)
		α = 69.039(3) β = 88.500(4) γ = 68.036(3)
	C2 (21.23 %)	a = 7.6404(7) b = 8.1244(9) c = 10.116(1)
		α = 90.00 β = 110.66(1) γ = 90.00
	M (11.41 %)	a = 6.611(1) b = 8.314(2) c = 9.483(1)
		α = 90.00 β = 100.54(2) γ = 90.00
1.00	CT (23.62 %)	a = 5.1925(3) b = 5.3492(3) c = 6.5674(3)
		α = 68.997(5) β = 88.449(6) γ = 67.980(5)
	C2 (19.24 %)	a = 7.6147(5) b = 8.1294(6) c = 10.1159(6)
		α = 90.00 β = 110.374(6) γ = 90.00
	MO (18.80 %)	a = 6.0533(5) b = 11.098(1) c = 8.4009(8)
		α = 90.00 β = 90.00 γ = 90.00
	M (26.46 %)	a = 6.5954(4) b = 8.3555(4) c = 9.4997(6)
		α = 90.00 β = 100.520(5) γ = 90.00
	CM (13.71 %)	a = 6.1842(7) b = 8.084(1) c = 6.3181(9)
		α = 90.00 β = 111.38(1) γ = 90.00
1.50	CM (100 %)	a = 6.3729(1) b = 8.1626(2) c = 6.1790(1)
		α = 90.00 β = 115,608(2) γ = 90.00
2.00	CM (73.01 %)	a = 6.5984(3) b = 8.1901(3) c = 6.1459(3)
		α = 90.00 β = 119.656(2) γ = 90.00
	MO (26.99 %)	a = 6.0787(3) b = 11.4674(8) c = 8.2078(5)
		α = 90.00 β = 90.00 γ = 90.00
2.50	MO (92.55 %)	a = 6.0975(1) b = 11.4523(2) c = 8.2555(2)
		α = 90.00 β = 90.00 γ = 90.00
3.00	MO (80.94 %)	a = 6.0613(3) b = 11.4395(5) c = 8.3115(4)
		α = 90.00 β = 90.00 γ = 90.00
	M (19.06 %)	a = 6.6051(5) b = 8.4106(6) c = 9.4815(8)
		α = 90.00 β = 100.626(8) γ = 90.00

Crystalline phases: CT = Cu_3_V_2_O_8_ (triclinic), CM = Cu_3_V_2_O_8_ (monoclinic), MO = Mg_3_V_2_O_8_ (orthorhombic), C1 = α-Cu_2_V_2_O_7_ (orthorhombic), C2 = β-Cu_2_V_2_O_7_ (monoclinic), M = α-Mg_2_V_2_O_7_ (monoclinic).

**Table 5:** **Unit cell parameters in crystalline phases with weight fractions higher than 8% from Mg**_**x**_ **Cu**_**3-x**_ **V**_**2**_ **O**_**8**_ **(0 ≤ x ≤ 3) compositions fired at 800°C/1 h**

**x**	**Crystalline phases (weight fractions)**	**Unit cell parameters (Å and degrees)**
1.00	MC (100%)	a = 6.4444 (1) b = 8.2992 (1) c = 11.4842 (2)
		α = 90.00 β = 90.61 γ = 90.00
1.50	CM (55.49%)	a = 6.3869 (3) b = 8.1575 (4) c = 6.1600 (3)
		α = 90.00 β = 116.308 (3) γ = 90.00
	MC (40.82%)	a = 6.4472 (1) b = 8.2960 (2) c = 11.4933 (2)
		α = 90.00 β = 90.479 (2) γ = 90.00
2.00	CM (100%)	a = 6.6120 (1) b = 8.1901 (1) c = 6.1525 (1)
		α = 90.00 β = 119.795 (1) γ = 90.00
2.50	MO (92.55%)	a = 6.0963 (1) b = 11.4581 (2) c = 8.2556 (1)
		α = 90.00 β = 90.00 γ = 90.00
3.00	MO (76.55%)	a = 6.06485 (6) b = 11.4416 (1) c = 8.31576 (8)
		α = 90.00 β = 90.00 γ = 90.00
	MT (23.45%)	a = 4.9304 (2) b = 5.4199 (2) c = 10.6787 (4)
		α = 100.321 (3) β = 102.808 (3) γ = 98.640 (3)

Crystalline phases: CM = Cu_3_V_2_O_8_ (monoclinic), MC = MgCu_2_V_2_O_8_ (monoclinic), MO = Mg_3_V_2_O_8_ (orthorhombic), C2 = β-Cu_2_V_2_O_7_ (monoclinic), MT = Mg_2_V_2_O_7_ (triclinic).

**Table 6:** **Unit cell parameters in crystalline phases with weight fractions higher than 8% from Mg**_**x**_ **Cu**_**3-x**_ **V**_**2**_ **O**_**8**_ **(0 ≤ x ≤ 3) compositions fired at 1000°C/1 h**

**x**	**Crystalline phases (weight fractions)**	**Unit cell parameters (Å and degrees)**
1.00	MO (84.55%)	a = 6.0999 (2) b = 11.4633 (4) c = 8.2642 (3)
		α = 90.00 β = 90.90 γ = 90.00
	CM (12.12%)	a = 6.4805 (9) b = 8.458 (1) c = 6.2031 (8)
		α = 90.00 β = 117.29 (1) γ = 90.00
1.50	MO (66.10%)	a = 6.0850 (2) b = 11.4556 (4) c = 8.2858 (2)
		α = 90.00 β = 90.90 γ = 90.00
	CM (17.35%)	a = 6.5138 (7) b = 8.590 (1) c = 6.2116 (8)
		α = 90.00 β = 117.495 (9) γ = 90.00
	M (16.54%)	a = 6.5786 (6) b = 8.4263 (7) c = 9.5107 (9)
		α = 90.00 β = 100.49 (1) γ = 90.00
2.00	MO (78.31%)	a = 6.0826 (1) b = 11.4538 (3) c = 8.2864 (2)
		α = 90.00 β = 90.00 γ = 90.00
	M (9.13%)	a = 6.1867 (6) b = 8.2179 (7) c = 6.3129 (5)
		α = 90.00 β = 112.016 (8) γ = 90.00
2.50	MO (96.02%)	a = 6.0775 (1) b = 11.4473 (2) c = 8.2813 (1)
		α = 90.00 β = 90.00 γ = 90.00
3.00	MO (77.73%)	a = 6.06118 (5) b = 11.4365 (1) c = 8.31278 (7)
		α = 90.00 β = 90.00 γ = 90.00
	MT (22.27%)	a = 4.9278 (2) b = 5.4169 (2) c = 10.6733 (4)
		α = 100.326 (2) β = 102.779 (3) γ = 98.632 (2)

Crystalline phases: CM = Cu_3_V_2_O_8_ (monoclinic), MO = Mg_3_V_2_O_8_ (orthorhombic), M = Mg_2_V_2_O_7_ (monoclinic), MT = Mg_2_V_2_O_7_ (triclinic).

**Table 7:** **Unit cell parameters in crystalline phases with weight fractions higher than 8% from Mg**_**y**_ **Cu**_**2-y**_ **V**_**2**_ **O**_**7**_ **(0 ≤ y ≤ 2) compositions fired at 600°C/12 h**

**y**	**Crystalline phases (weight fractions)**	**Unit cell parameters (Å and degrees)**
		**a / b / c // α / β / γ**
0.000	C1 (100%)	8.40244 (8) / 20.6701 (2) / 6.44418 (5) // 90.00 / 90.00 / 90.00
0.250	C2 (87.6%)	7.67549 (8) / 8.0734 (1) / 10.1196 (2) // 90.00 / 110.437 (1) / 90.00
	C1 (12.4%)	8.3703 (3) / 20.6942 (8) / 6.4551 (3) // 90.00 / 90.00 / 90.00
0.500	C2 (63.1%)	7.6554 (1) / 8.1002 (1) / 10.1168 (2) // 90.00 /110.550 (1) / 90.00
	C2’(26.8%)	7.6219 (4) / 8.0881 (6) / 10.1139 (6) // 90.00 / 110.655 (5) / 90.00
	M3 (10.1%)	6.7623 (6) / 8.4212 (7) / 9.3331 (9) // 90.00 / 95.61 (1) / 90.00
0.750	M3 (50.51%)	6.7701 (5) / 8.4666 (5) / 9.3134 (7) // 90.00 / 96.577 (7) / 90.00
	C2’(29.1%)	7.6322 (4) / 8.0720 (5) / 10.1722 (7) // 90.00 / 111.164 (5) / 90.00
	C2 (20.4%)	7.6410 (4) / 8.1202 (5) / 11.1141 (5) // 90.00 / 110.629 (5) / 90.00
1.000	M3 (54.4%)	6.8418 (4) / 8.4116 (4) / 9.3644 (5) // 90.00 / 95.904 (5) / 90.00
	C2 (45.6%)	7.6091 (4) / 8.0465 (3) / 10.1275 (5) // 90.00 / 110.048 (5) / 90.00
1.250	C2 (36.4%)	7.6115 (2) / 8.0629 (2) / 10.1148 (3) // 90.00 / 109.804 (3) / 90.00
	M4 (40.0%)	6.8263 (2) / 8.0816 (3) / 9.3090 (3) // 90.00 / 88.438 (3) / 90.00
	M3 (23.6%)	6.8846 (3) / 8.4130 (4) / 9.2537 (5) // 90.00 / 95.778 (4) / 90.00
1.500	C2 (37.4%)	7.5684 (3) / 8.0539 (3) / 10.1486 (4) // 90.00 / 109.879 (4) / 90.00
	M4 (42.3%)	6.8291 (2) / 8.0591 (3) / 9.3291 (3) // 90.00 / 88.249 (3) / 90.00
	M1 (20.3%)	6.5895 (5) / 8.3913 (6) / 9.4963 (7) // 90.00 / 100.397 (7) / 90.00
1.750	M1 (73.1%)	6.58498 (9) / 8.3914 (1) / 9.4872 (1) // 90.00 / 100.462 (1) / 90.00
	M4 (15.1%)	6.8308 (4) / 8.0534 (4) / 9.3113 (5) // 90.00 / 88.705 (6) / 90.00
	C2 (11.8%)	7.5662 (5) / 8.0592 (5) / 10.1427 (6) // 90.00 / 109.864 (6) / 90.00
2.000	M1 (83.9%)	6.60534 (6) / 8.41437 (8) / 9.48264 (9) // 90.00 / 100.6104 (8) / 90.00
	M2 (16.1%)	6.6639 (2) / 8.4361 (3) / 9.5745 (3) // 90.00 / 99.446 (3) / 90.00

Crystalline phases: C1 = α-Cu_2_V_2_O_7_, C2 = β-Cu_2_V_2_O_7_ (monoclinic, C2: β < 110.6°, C2’: β > 110.6°), M = α-Mg_2_V_2_O_7_ (monoclinic, M1: β = 100.0-100.6°, M2: β = 98.0-99.6°, M3: β = 94.5-97.0°, M4: β = 88.0-92.0°).

**Table 8:** **Unit cell parameters in crystalline phases with weight fractions higher than 8% from Mg**_**y**_ **Cu**_**2-y**_ **V**_**2**_ **O**_**7**_ **(0 ≤ y ≤ 2) compositions fired at 800°C/1 h**

**y**	**Crystalline phases (weight fractions)**	**Unit cell parameters (Å and degrees)**
		**a / b / c // α / β / γ**
0.500	C2 (56.8%)	7.6430 (3) / 8.1076 (3) / 10.1147 (4) // 90.00 / 110.589 (4) / 90.00
	M4 (26.3%)	6.719 (1) / 8.553 (1) / 9.846 (2) // 90.00 / 91.33 (2) / 90.00
	M2 (16.9%)	6.936 (1) / 8.423 (1) / 9.793 (2) // 90.00 / 98.32 (1) / 90.00
0.750	M3 (48.4%)	6.9060 (4) / 8.3131 (5) / 9.5450 (5) // 90.00 / 95.346 (7) / 90.00
	C2 (36.0%)	7.6308 (4) / 8.1127 (5) / 10.0542 (7) // 90.00 / 110.389 (6) / 90.00
	C3 (15.1%)	4.9809 (5) / 5.8341 (6) / 10.933 (1) // 101.554 (8) / 96.517 (7) / 95.032 (8)
1.000	M4 (91.3%)	6.7766 (4) / 8.4899 (4) / 9.1447 (6) // 90.00 / 88.294 (5) / 90.00
	C2 (8.7%)	7.542 (2) / 8.083 (2) / 10.0574 (2) // 90.00 / 110.59 (2) / 90.00
1.250	M4 (79.9%)	6.81447 (7) / 8.11262 (8) / 9.30424 (9) // 90.00 / 88.444 (1) / 90.00
	C2 (13.9%)	7.6192 (3) / 8.0576 (4) / 10.1113 (4) // 90.00 / 109.689 (4) / 90.00
1.500	C1 (63.8%)	8.3392 (1) / 20.8256 (3) / 6.44642 (9) // 90.00 / 90.00 / 90.00
	M4 (32.1%)	6.7458 (3) / 7.8414 (3) / 9.2234 (3) // 90.00 / 90.890 (3) / 90.00
1.750	M4 (59.3%)	6.7825 (3) / 8.0761 (3) / 9.3084 (4) // 90.00 / 89.448 (4) / 90.00
	MT (40.7%)	4.9343 (4) / 5.4102 (5) / 10.7164 (9) // 99.866 (7) / 102.996 (5) / 98.743 (7)
2.000	M4 (59.3%)	6.7823 (1) / 8.0669 (2) / 9.3201 (2) // 90.00 / 89.345 (2) / 90.00
	MT (40.7%)	4.9354 (2) / 5.4094 (2) / 10.7145 (5) // 99.874 (3) / 102.959 (3) / 98.756 (3)

Crystalline phases: C1 = α-Cu_2_V_2_O_7_, C2 = β-Cu_2_V_2_O_7_ (monoclinic, C2: β < 110.6°, C2’: β > 110.6°), C3 = γ-Cu_2_V_2_O_7_ (triclinic), M = α-Mg_2_V_2_O_7_ (monoclinic, M1: β = 100.0-100.6°, M2: β = 98.0-99.6°, M3: β = 94.5-97.0°, M4: β = 88.0-92.0°), MT = γ-Mg_2_V_2_O_7_ (triclinic).

**Table 9:** **Unit cell parameters in crystalline phases with weight fractions higher than 8% from Mg**_**y**_ **Cu**_**2-y**_ **V**_**2**_ **O**_**7**_ **(0 ≤ y ≤ 2) compositions fired at 1000°C/1 h**

**y**	**Crystalline phases weight fractions)**	**Unit cell parameters (Å and degrees)**
		**a / b / c // α / β / γ**
1.500	MT (41.6%)	4.9331 (2) / 5.4122 (3) / 10.7005 (5) // 100.037 (4) / 102.907 (3) / 98.777 (4)
	M1 (39.7%)	6.6011 (4) / 8.4011 (4) / 9.5027 (5) // 90.00 / 100.394 (5) / 90.00
1.750	M1 (81.1%)	6.5951 (1) / 8.4057 (2) / 9.5075 (2) // 90.00 / 100.427 (2) / 90.00
	MT (16.5%)	4.9318 (4) / 5.4130 (5) / 10.7219 (9) // 100.109 (8) / 102.908 (6) / 98.769 (7)
2.000	MT (69.0%)	4.9276 (2) / 5.4164 (2) / 10.6697 (4) // 100.318 (2) / 102.785 (3) / 98.617 (3)
	MO (17.4%)	6.0832 (4) / 10.8818 (5) / 8.6212 (5) // 90.00 / 90.00 / 90.00
	M1 (13.6%)	6.4596 (7) / 8.5135 (8) / 9.403 (1) // 90.00 / 102.00 (1) / 90.00

Crystalline phases: M = α-Mg_2_V_2_O_7_ (monoclinic, M1: β = 100.6-102.0^o^), MT = γ-Mg_2_V_2_O_7_ (triclinic), MO = Mg_3_V_2_O_8_ (orthorhombic).

From Mg_x_Cu_3-x_V_2_O_8_ compositions solid solutions with triclinic Cu_3_V_2_O_8_ structure are obtained when 0.0 ≤ x ≤ 1.0 at 600°C. At this temperature, the c unit cell parameter increases slightly with the replacement of Cu (II) ion by a slightly smaller one (Mg (II)) from Mg_x_Cu_3-x_V_2_O_8_ compositions when 0.0 ≤ x ≤ 1.0. Ionic radii values do not explain this fact. A slight contraction of unit cell is expected. Structural distortion might explain the c increase with incorporation of Mg (II) ions in a structure (Tena 2012).

Variation of unit cell parameters obtained from Mg_x_Cu_3-x_V_2_O_8_ (x ≥ 1.0) compositions fired at 800°C is noticeable in monoclinic Cu_3_V_2_O_8_ structure (detected when 1.5 ≤ x ≤ 2.0) and it indicates the formation of solid solutions with monoclinic Cu_3_V_2_O_8_ structure in 1.5 ≤ x ≤ 2 compositional range at 600°C and 800°C.

**Figure 4:**
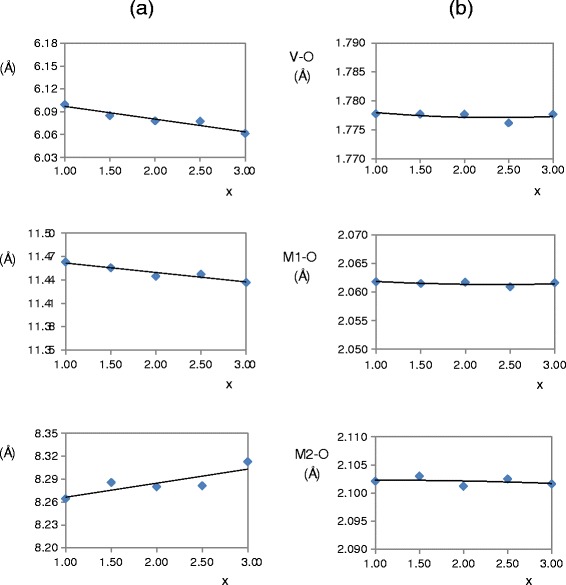
**Cell parameters in the Mg**_**3**_ **V**_**2**_ **O**_**8**_ **structure.** Variation of unit cell parameters **(a)** and interatomic distances **(b)** in Mg orthovanadate structure with composition obtained from Mg_x_Cu_3-x_V_2_O_8_ samples fired at 1000°C/1 h.

Solid solutions with orthorhombic Mg_3_V_2_O_8_ structure are also obtained when 2.5 ≤ x ≤ 3.0 at 600, 800°C and when 1.0 ≤ x ≤ 3.0 at 1000°C. Figure 4 shows unit cell parameters and interatomic distances in Mg orthovanadate structure (orthorhombic symmetry) obtained from Mg_x_Cu_3-x_V_2_O_8_ samples fired at 1000°C/1 h. At 1000°C, the a and b unit cell parameters decrease with the replacement of Cu (II) ion by a slightly smaller one (Mg (II)). Average changes of interatomic distances are very slight. Changes in intensities with composition (Figure 2) are due to changes in both the atomic coordinates and the Mg/Cu ratio in these sites in orthorhombic Mg_3_V_2_O_8_ structure when solid solutions are formed. These solid solutions are the most stable solid solutions obtained in this study from Mg_x_Cu_3-x_V_2_O_8_ compositions.

From unit cell parameters values obtained in Mg_y_Cu_2-y_V_2_O_7_ compositions, the formation of two kinds of solid solutions with monoclinic β-Cu_2_V_2_O_7_ and monoclinic α-Mg_2_V_2_O_7_ structures can be confirmed. Two crystalline phases with the same structure (β-Cu_2_V_2_O_7_ or α-Mg_2_V_2_O_7_) are detected in some samples (Tables 7 and 8). This fact indicates that the composition of these two crystalline phase (C2: β < 110.6 ^o^ and C2’: β > 110.6° with β-Cu_2_V_2_O_7_ structure or two of M1: β = 100.0-100.6°, M2: β = 98.0-99.6°, M3: β = 94.5-97.0°, M4: β = 88.0-92.0° phases with α-Mg_2_V_2_O_7_ structure) is slightly different at 600 and 800°C in the conditions of this study. This slight difference between the two crystalline phases with the same structure is evidenced with differences in unit cell parameters values obtained. In Mg_y_Cu_2-y_V_2_O_7_ compositions fired at 600°C, the values of a and β parameters decrease when y increases in β-Cu_2_V_2_O_7_ structure (crystalline phase is obtained with a weight fraction > 35% when 0.25 ≤ y ≤ 1.5). At 800°C this crystalline phase is obtained with a weight fraction > 35% only when 0.5 ≤ y ≤ 0.75. In crystalline phase with α-Mg_2_V_2_O_7_ structure, β angle value is close to 100° at 300°C and it is close to 90° at 800°C. When two crystalline phases with this structure are detected in the same sample, unit cell parameters are 0.2 Å (a, b, c parameters) or 8 degrees (β parameter) longer and wider in one of them. This fact is in accordance with the formation of solid solutions in this monoclinic α-Mg_2_V_2_O_7_ structure with the replacement of Mg (II) ion by Cu (II) and with an important distortion structural in these formed solid solutions. Solid solutions with monoclinic α-Mg_2_V_2_O_7_ structure are obtained when 0.5 ≤ y ≤ 2.0 at 600 and 800°C. At 1000°C, these last solid solutions are obtained when y ≥ 1.5, including all unmelted samples (Table 9).

Coordination number of V (V) ion in Cu and Mg orthovanadate and divanadate structures is four. Coordination number of Cu (II) ion is four and five and coordination number of Mg (II) ion is six in these structures. Values obtained from samples are in accordance with literature about these structures. In distorted monoclinic structure of Cu_3_V_2_O_8_ obtained from Mg_2_CuV_2_O_8_ composition at 800°C, the coordination number of Cu (II) and Mg (II) ions are also six.

**Figure 5:**
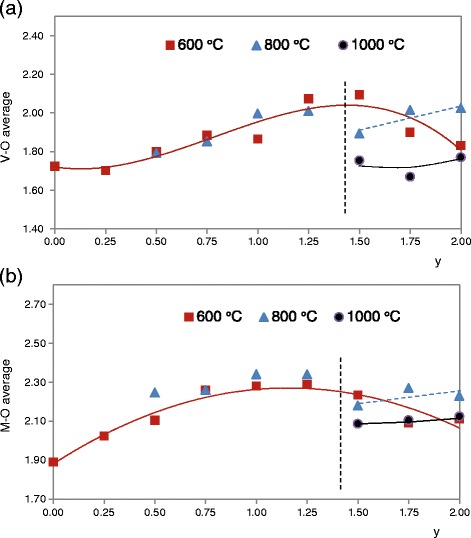
**Interatomic distances in Mg**_**y**_ **Cu**_**2-y**_ **V**_**2**_ **O**_**7**_ **compositions.** Average interatomic V-O **(a)** and M-O **(b)** distances in Mg_y_Cu_2-y_V_2_O_7_ (0 ≤ y ≤ 1) compositions fired at 600°C/12 h, 800°C/1 h and 1000°C/1 h considering weight fraction of crystalline phases.

Average interatomic V-O distances is smaller than average interatomic Cu-O or Mg-O distances in all of detected crystalline phases. Figure 5 shows the average interatomic V-O and Cu-O or Mg-O distances obtained from Mg_y_Cu_2-y_V_2_O_7_ compositions considering all the crystalline phases in each composition and its weight fraction in samples fired at 600, 800 and 1000°C. These M-O (M = Cu, Mg) interatomic distances increase with magnesium amount (y) when 0 ≤ y ≤ 1.25. This increased average M-O distance is coincident with destabilization of structures of Cu divanadate and a change is observed when y > 1.25. Structures of Cu divanadate are unstable with temperature and structures of Mg divanadates are stable at 1000°C when 1.5 ≤ y ≤ 2.0 (Table 3).

**Figure 6:**
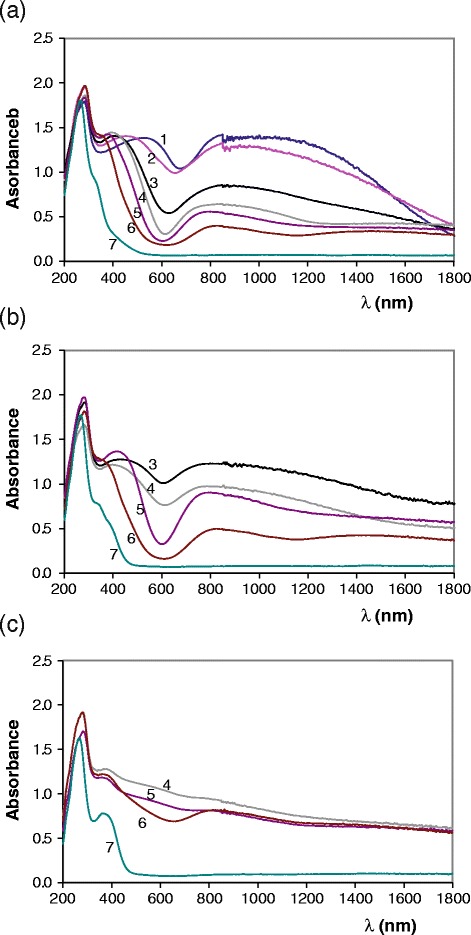
**Variation of UV–vis-NIR spectra from Mg**_**x**_ **Cu**_**3-x**_ **V**_**2**_ **O**_**8**_ **compositions.** UV–vis-NIR spectra of Mg_x_Cu_3-x_V_2_O_8_ (0.0 ≤ x ≤ 3.0) samples fired at: **(a)** 600, **(b)** 800 and **(c)** 1000 ºC; x = 0.0 (1), x = 0.5 (2), x = 1.0 (3), x = 1.5 (4), x = 2.0 (5), x = 2.5 (6) and x = 3.0 (7).

**Figure 7:**
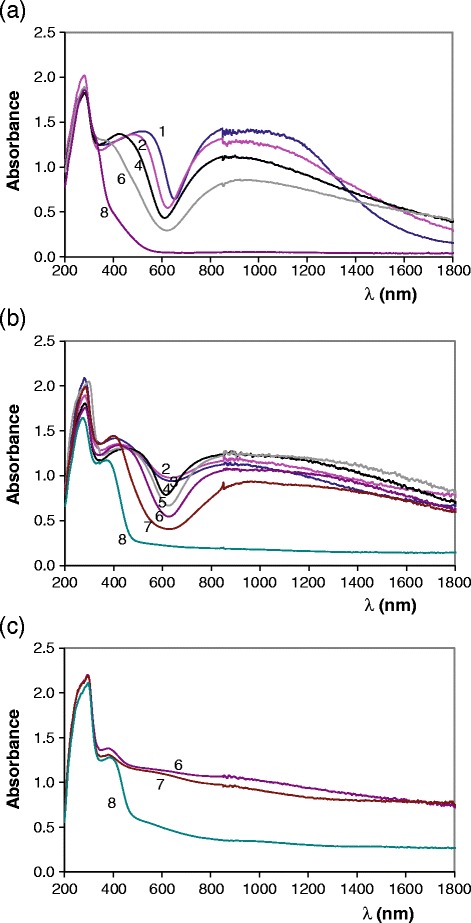
**Variation of UV–vis-NIR spectra from Mg**_**y**_ **Cu**_**2-y**_ **V**_**2**_ **O**_**7**_ **compositions.** UV–vis-NIR spectra of Mg_y_Cu_2-y_V_2_O_7_ (0.0 ≤ y ≤ 2.0) samples fired at : **(a)** 600, **(b)** 800 and **(c)** 1000 ºC; y = 0.0 (1), y = 0.50 (2), y = 0.75 (3), y = 1.0 (4), y = 1.25 (5), y = 1.50 (6), y = 1.75 (7) and y = 2.0 (8).

**Figure 8:**
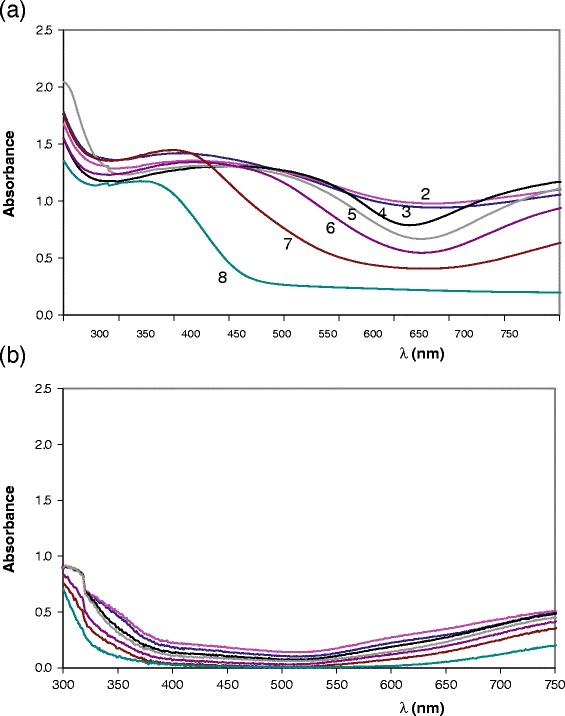
**Visible spectra of compositions at 800°C.** Visible spectra of: **(a)** Mg_y_Cu_2-y_V_2_O_7_ (0.0 ≤ y ≤ 2.0) and **(b)** Mg_x_Cu_2-x_P_2_O_7_ (0.0 ≤ x ≤ 1.5) samples fired at 800°C; y = 0.50 (2), y = 0.75 (3), y = 1.00 (4), y = 1.25 (5), y = 1.50 (6), y = 1.75 (7), y =2.00 (8).

Figures 6 and 7 show UV–vis-NIR spectra of Mg_x_Cu_3-x_V_2_O_8_ (0 ≤ x ≤ 3) and Mg_y_Cu_2-y_V_2_O_7_ (0 ≤ y ≤ 2) compositions fired at 600, 800 and 1000°C. Visible spectra obtained from Mg_y_Cu_2-y_V_2_O_7_ (0 ≤ y ≤ 2) compositions fired at 800°C/1 h and Mg_x_Cu_2-x_P_2_O_7_ (0 ≤ x ≤ 1.5) compositions fired at 800°C/5d (Tena 2012) are shown in Figure 8. Most of complex with Cu (II) ion are formed with four ligands (IC = 4). The reason for the unusual behaviour is connected with the Jahn-Teller effect. Because of that, the Cu (II) ions do not bind the fifth and sixth ligands strongly. Structural distortion due to this Jahn-Teller effect can explain the asymmetry in observed bands. From Cu (II) ion (d^9^) a transition d-d is allowed. Experimentally electronic spectra of Cu (II) ion are often characterized by a single highly asymmetric band. In this study spectra show a strong absorbance in 700–1400 nm wavelength range with the absorption maximum at 800–900 nm. It can be associated with Cu (II) d-d transition. Bands due to d-d transitions are not expected from V (V) ion. Strong absorbance in 430–600 nm wavelength range (in visible wavelength range) with the absorption maximum depending of copper amount in the sample (x or y values) is detected in Mg_x_Cu_3-x_V_2_O_8_ (0 ≤ x ≤ 3) and Mg_y_Cu_2-y_V_2_O_7_ (0 ≤ y ≤ 2) compositions at 300°C/12 h, 600°C/12 h, 800°C/1 h and 1000°C/1 h. The strong absorbance in 350–600 nm wavelength range is not observed in Mg_x_Cu_2-x_P_2_O_7_ compositions (Figure 8) and in Mg_x_Cu_3-x_V_2_O_8_ (x = 3) and Mg_y_Cu_2-y_V_2_O_7_ (y = 2) compositions. It is associated with charge transfer between Cu-O in orthovanadates and divanadates when 0 ≤ x < 3 or when 0 ≤ y < 2. When x = 3 or y = 2, the charge transfer is associated with V-O charge transfer (λ < 430 nm in studied temperature range). Maximum of this band is observed at higher wavelength when copper amount is hight than when the copper amount is low. This strong absorbance in visible wavelength range explains the colour of these materials. At 600 and 800°C, coloration from samples with vanadate structures is red-brown, orange and yellow and is very different to the weak blue coloration obtained from samples with phosphate structures (Tena 2012). At 1000°C, brown-dark and black colorations are obtained when x < 3.0 or y < 2.0 and a strong absorbance in 400–1800 nm range is obtained in spectra. In the most of samples band due to d-d transition and band due to charge transfer can not be differentiated at 1000°C. Vanadate structure does not seem to be the only important factor for the presence of this charge transfer band because it is present in Mg_x_Cu_3-x_V_2_O_8_ (x ≠ 3) compositions and in Mg_x_Cu_2-y_V_2_O_7_ (y ≠ 2) compositions with different crystalline phases detected by XRD (Tables 2 and 3). The difference of coloration of Cu phosphates and Cu vanadates might be explained from differences in interatomic Cu-O distances due to the presence of bonds P-O or V-O. Interatomic Cu-O distances are smaller in vanadate structures than in phosphate structures (Cu-O bond is more strong in vanadates than in phosphates) because the V-O bond is weaker than the P-O bond (interatomic V-O distances are slightly greater than interatomic P-O distances). Table 10 shows interatomic distances in some of these compositions.

**Table 10:** **Interatomic distances in orthovanadates, divanadates and diphosphates of Cu and Mg**

**Composition**	**Mg** ^**2+**^ **-O (Å)**	**Cu** ^**2+**^ **-O (Å)**	**V-O or P-O (Å)**
^1^ Cu_3_V_2_O_8_	-----	1.912 (4) - 1.988 (5)	1.643 (3) - 1.795 (4)
^1^ Cu_3_P_2_O_8_	-----	1.925 (3) - 1.981 (3)	1.510 (2) - 1.572 (2)
^1^ Mg_3_V_2_O_8_	2.022 (1) - 2.135 (1)	-----	1.695 (1) - 1.809 (1)
^1^ Mg_3_P_2_O_8_	1.965 (8) - 2.142 (7)	-----	1.527 (5) - 1.535 (8)
^2^ Mg_x_Cu_3-x_V_2_O_8_	1.880 (2) - 2.306 (1)		1.570 (3) -1.972 (2)
^1^ Cu_2_V_2_O_7_	-----	1.880 (5) - 2.542 (4)	1.644 (7) - 1.770 (4)
^1^ Cu_2_P_2_O_7_	-----	1.929 (1) - 2.313 (1)	1.437 (1) - 1.561 (1)
^1^ Mg_2_V_2_O_7_	1.992 (3) - 2.221 (3)	-----	1.629 (3) - 1.817 (4)
^1^ Mg_2_P_2_O_7_	1.850 (1) - 2.381 (1)	-----	1.543 (1) - 1.785 (1)
^2^ Mg_y_Cu_2-y_V_2_O_7_	1.862 (3) - 2.282 (1)		1.620 (2) - 1.904 (4)
^3^ Mg_x_Cu_2-x_P_2_O_7_	1.927 (3) - 2.282 (2)		1.495 (2) - 1.574 (1)

^1^ Inorganic Crystal Structure Database (ICSD) (2013).

^2^ Mg_x_Cu_3-x_V_2_O_8_ (0 ≤ x ≤ 3) and Mg_y_Cu_2-y_V_2_O_7_ (0 ≤ y ≤ 2) compositions prepared in this study.

^3^ (Tena 2012).

CIE L*, a* and b* parameters of Mg_x_Cu_3-x_V_2_O_8_ (0 ≤ x ≤ 3) and Mg_y_Cu_2-y_V_2_O_7_ (0 ≤ y ≤ 2) samples fired at 600, 800 and 1000°C are shown in Table 11.

**Table 11:** CIE L* a* b* colour parameters in Mg_**x**_ **Cu**_**3-x**_ **V**_**2**_ **O**_**8**_ **(0 ≤ x ≤ 3) and Mg**_**y**_ **Cu**_**2-y**_ **V**_**2**_ **O**_**7**_ **(0 ≤ y ≤ 2) samples**

**x**	**Observed colour**	**Mg**_**x**_ **Cu**_**3-x**_ **V**_**2**_ **O**_**8**_ **samples fired at 600°C/12 h**		
		L*	a*	b*
0.00	dark brown	41.57	4.10	0.79
0.50	dark brown	43.59	5.83	6.82
1.00	orange	56.94	13.29	25.79
1.50	yellow	66.67	18.29	42.22
2.00	yellow	76.77	6.47	47.76
2.50	light brown	83.12	- 1.34	35.49
3.00	light yellow	94.76	- 2.00	13.35
**y**	**Observed colour**	**Mg**_**y**_ **Cu**_**2-y**_ **V**_**2**_ **O**_**7**_ **samples fired at 600°C/12 h**		
		L*	a*	b*
0.00	dark brown	43.33	13.86	4.91
0.25	red-brown	38.34	20.54	9.80
0.50	red-brown	50.60	21.72	16.23
0.75	orange	46.88	25.71	29.13
1.00	orange	58.96	19.05	30.90
1.25	orange	57.06	19.77	46.16
1.50	brown	70.76	10.83	36.19
1.75	light brown	69.70	4.76	34.91
2.00	light yellow	92.46	- 1.35	22.95
**x**	**Observed colour**	**Mg**_**x**_ **Cu**_**3-x**_ **V**_**2**_ **O**_**8**_ **samples fired at 800°C/1 h**		
		L*	a*	b*
1.00	dark brown	46.73	3.03	6.73
1.50	brown	53.37	4.77	15.20
2.00	yellow	68.97	12.02	41.84
2.50	light yellow	83.96	0.38	39.86
3.00	light yellow	95.50	- 3.57	11.54
**y**	**Observed colour**	**Mg**_**y**_ **Cu**_**2-y**_ **V**_**2**_ **O**_**7**_ **samples fired at 800°C/1 h**		
		L*	a*	b*
0.50	dark brown	48.90	4.19	10.50
0.75	dark brown	47.58	3.95	8.75
1.00	brown	49.18	10.31	11.09
1.25	orange	52.11	13.11	16.37
1.50	orange	57.13	13.67	24.55
1.75	light brown	69.05	3.87	35.70
2.00	light brown	85.00	- 5.37	22.14
**x**	**Observed colour**	**Mg**_**x**_ **Cu**_**3-x**_ **V**_**2**_ **O**_**8**_ **samples fired at 1000°C/1 h**		
		L*	a*	b*
1.00	grey	52.75	0.96	0.56
1.50	dark brown	48.74	1.15	4.58
2.00	dark brown	51.37	1.46	6.09
2.50	brown	58.02	2.69	12.51
3.00	yellow	94.28	- 4.86	17.95
**y**	**Observed colour**	**Mg**_**y**_ **Cu**_**2-y**_ **V**_**2**_ **O**_**7**_ **samples fired at 1000°C/1 h**		
		L*	a*	b*
1.50	black	46.85	- 0.16	3.84
1.75	black-brown	47.39	0.31	3.86
2.00	yellow-brown	67.25	- 1.12	20.11

Interesting yellow colorations are obtained when x = 1.5 and 2.0 at 600°C/12 and when x = 2.0 at 800°C/1 h. In these samples that development yellow colorations, crystalline phase with monoclinic Cu_3_V_2_O_8_ structure is detected. The best yellow colour is obtained from Mg_2_CuV_2_O_8_ (x = 2.0) solid solution with monoclinic Cu_3_V_2_O_8_ structure. This is the only crystalline phase detected at 800°C in conditions of this study. The Mg (II) incorporated into this structure stabilizes this crystalline phase at temperatures higher than 780°C (melting point of Cu_3_V_2_O_8_). In Mg_2_CuV_2_O_8_ composition fired at 800°C, average distances of M1-O (M1 = Cu, Mg) = 1.9587 Å and M2-O (M2 = Cu, Mg) = 2.1129 Å are obtained. Yellow colorations are not obtained from Cu, Mg divanadates.

The colour red-brown obtained from Mg_0.5_Cu_1.5_V_2_O_7_ (y = 0.5) fired at 600°C /12 h is the most noticeable colour but it is unstable at 800°C /1 h. Dark brown colour is obtained from this composition at 800°C. The major crystalline phase detected by XRD is the crystalline phase with β-Cu_2_V_2_O_7_ structure in this sample fired at 600°C.

Orange colour is obtained when y = 0.75, 1.00 and 1.25 at 600°C/12 h and when y = 1.25 and 1.50 at 800°C/1 h. In these orange materials, a mixture of monoclinic crystalline phases with β-Cu_2_V_2_O_7_ and Cu_3_V_2_O_8_ or β-Cu_2_V_2_O_7_ and α-Mg_2_V_2_O_7_ structures is detected by XRD.

**Figure 9:**
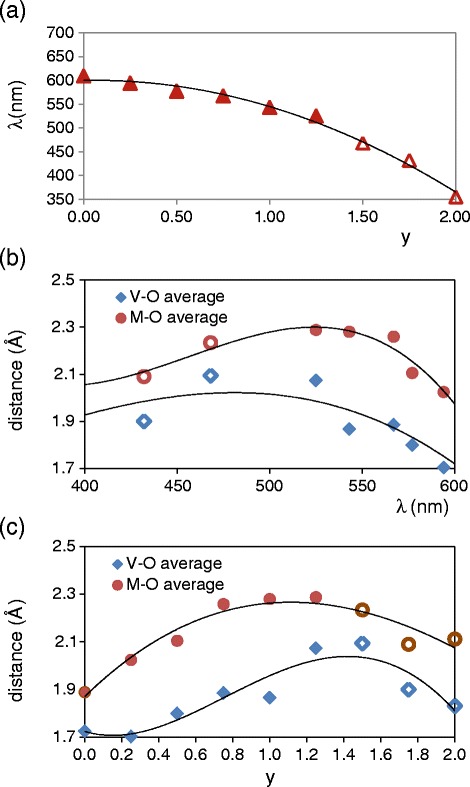
**Variation of interatomic distances with wavelength and composition.** Variation of wavelength of Cu-O charge transfer with composition **(a)**, variation of average interatomic V-O and M-O (M = Cu, Mg) distances with wavelength **(b)** and variation of these average interatomic distances with composition **(c)** from Mg_y_Cu_2-y_V_2_O_7_ samples fired at 600°C. Solid marks: y < 1.5; Empty marks: y ≥ 1.5.

Figure 9 shows the wavelength of charge transfer in Mg_y_Cu_2-y_V_2_O_7_ compositions fired at 600°C/12 h (bands in Figure 7 (a)) and the variation of average V-O and M-O distances with wavelength of charge transfer and with composition (y). Inflection point between maximum and minimum absorbance in band is considered in assignation of wavelength values. Distances are calculated considering all the crystalline phases detected by XRD in each composition and its weight fraction in this sample. When Cu (II) amount (y) decreases in samples, the wavelength of charge transfer decreases in all compositions at this temperature and colour of samples changes from red-brown to yellow. From variation of average interatomic distances at 600°C/12 h, the longest average M-O distances is detected when 0.75 ≤ y ≤ 1.25 and orange coloration is obtained in these materials (charge transfer about 550 nm). At this temperature red-brown coloration is obtained in samples with the shortest average M-O distances (charge transfer about 570 nm). The obtained colorations are different with similar average distances when Mg amount is high (y > 1.25) and when Cu amount is high (y < 1.25) because Mg (II) and Cu (II) radius are similar. Structural changes must be also considered to explain the complete evolution of colour with composition.

## Conclusions

From Mg_x_Cu_3-x_V_2_O_8_ compositions solid solutions with triclinic Cu_3_V_2_O_8_ structure are obtained in 0.0 ≤ x ≤ 1.0 at 600°C. When x = 1.0, MgCu_2_V_2_O_8_ compound is detected at 800°C. When x = 2.0, Mg_2_CuV_2_O_8_ compound with the ordered metal distributions is not detected in the conditions of this study. From this composition, solid solutions with monoclinic Cu_3_V_2_O_8_ structure (Cu1 in 2b sites) are obtained. These solid solutions with monoclinic Cu_3_V_2_O_8_ structure are obtained when 1.5 ≤ x ≤ 2.0 at 600°C and 800°C. Solid solutions with orthorhombic Mg_3_V_2_O_8_ structure are obtained when 2.5 ≤ x ≤ 3.0 at 600, 800°C and when 1.0 ≤ x ≤ 3.0 at 1000°C. In this study, the most stable solid solutions are obtained with Mg orthovanadate structure (orthorhombic).

From Mg_y_Cu_2-y_V_2_O_7_ compositions, the formation of two kind of solid solutions with β-Cu_2_V_2_O_7_ and α-Mg_2_V_2_O_7_ structures is detected. Solid solutions with monoclinic β-Cu_2_V_2_O_7_ structure are obtained when 0.25 ≤ y ≤ 1.50 at 600°C and when 0.50 ≤ y ≤ 0.75 at 800°C. Solid solutions with monoclinic α-Mg_2_V_2_O_7_ structure are obtained when 0.5 ≤ y ≤ 2.0 at 600 and 800°C and showing an important structural distortion. At 1000°C, solid solutions with α-Mg_2_V_2_O_7_ structure are obtained in 1.5 ≤ y ≤ 2.0 compositional range. It is proposed the existence of a new polymorph of Mg_2_V_2_O_7_ compound with α-Mn_2_V_2_O_7_ structure detected when y ≥ 1.25 at 300°C.

Strong absorbance in visible spectra is detected in Mg_x_Cu_3-x_V_2_O_8_ (0 ≤ x < 3) and Mg_y_Cu_2-y_V_2_O_7_ (0 ≤ y < 2) compositions which is associated with charge transfer between Cu-O in orthovanadates and divanadates. Wavelength of the abrupt change in absorbance is in accordance with Cu-O interatomic distances in these structures. In this study, the best yellow colour is obtained from Mg_2_CuV_2_O_8_ (x = 2.0) solid solution with monoclinic Cu_3_V_2_O_8_ structure. The colour red-brown is obtained from Mg_0.5_Cu_1.5_V_2_O_7_ (y = 0.5) fired at 600°C /12 h and it is unstable at 800°C /1 h. This red-brown colour is obtained when the average M-O distances are the shortest from divanadates. Orange colour is also obtained from some divanadates when average M-O distance is long.

Structural changes must be also considered to explain the colour of these materials. Thus, yellow colorations are obtained from orthovanadates and red-brown and orange colorations are obtained from divanadates.

## Additional files

Additional file 1:
**Information about crystal structures.**
Additional file 2:
**XRD patterns from Mg**_**x**_ **Cu**_**3-x**_ **V**_**2**_ **O**_**8**_ **compositions.**
Additional file 3:
**XRD patterns from Mg**_**y**_ **Cu**_**2-y**_ **V**_**2**_ **O**_**7**_ **compositions.**
