# Supplementary material for: Structural characterization and colour of MgxCu3-xV2O8 (0 ≤ x ≤ 3) and MgyCu2-yV2O7 (0 ≤ y ≤ 2) compositions
Source: Springerplus. 2015 Apr 3;4:163. doi: 10.1186/s40064-015-0908-8 (PMC4414859; doi:10.1186/s40064-015-0908-8)
Supplement: Additional file 1: — Information about crystal structures. [file 40064_2015_908_MOESM1_ESM.docx]

Cu_3_V_2_O_8_ and Mg_3_V_2_O_8_ structures

In Cu_3_V_2_O_8_ with PῙ space group there are two different crystallographic Cu(II) ions (Cu1, Cu2), only one crystallographic V(V) ion and four different crystallographic oxygen atoms (O1, O2, O3, O4). Cu1 ions are in 1a special sites, an inversion centre, and the rest are in 2i general sites. These ions (Cu1) are bonded at two O3 at
1.922(3) Å and at two O2 at 1.968(3) Å in a distorted plane square coordination. Cu2 ions are surrounded by five oxygen atoms in an irregular triangular bi-pyramidal coordination. The average distance Cu2-O is 2.015(3) Å. Thus, Cu2 has two short bonds with O3-Cu2-O4 angle of 169.5^o^ and the other three bonds are coplanar at right angles to them (Coing-Boyat 1982).

The structure of Cu_3_V_2_O_8_ compound with monoclinic symmetry and space group P12_1_/c1 is similar to the orthorhombic structure of Mg_3_V_2_O_8_ compound with space group Cmca. In these structures there are two different crystallographic M(II) ions M = Cu, Mg (M1, M2), only one crystallographic V(V) ion and four or three different crystallographic oxygen atoms (O1, O2, O3, O4 in Cu_3_V_2_O_8_ or O1, O2, O3 in Mg_3_V_2_O_8_). Cu1 ions are in 2b special sites and the rest are in 4e general sites in monoclinic Cu_3_V_2_O_8_ (P12_1_/c1). Mg1 are in 4a special sites, Mg2 are in 8e special sites, V1, O1 and O2 are in 8f special sites and O3 are in 16g general sites in orthorhombic Mg_3_V_2_O_8_ (Cmca). In the structure of MgCu_2_V_2_O_8_ (β = 90.44(2)ᵒ and Z = 4) and Mg_2_CuV_2_O_8_ (β = 116.22(3)ᵒ and Z = 2) compounds with monoclinic symmetry and space group P12_1_/c1, all the atoms are in 4e general sites except Cu1 in Mg_2_CuV_2_O_8_. In this last structure, Cu(II) ions (Cu1) are in 2a sites.

Mg_3_V_2_O_8_ structure is characterized by chains of edge-shared Mg2 octahedra running along the a axis. The network of octahedra containing M2 ions is linked by corner-sharing V-O tetrahedra. The VO_4_ tetrahedron is distorted with distances varying from 1.670(5) to 1.795(4) Å in Cu_3_V_2_O_8_ or 1.695(1) to 1.809(1) Å in Mg_3_V_2_O_8_. Average Cu-O bond length is 1.985(5) Å (1.945(5) to four Cu1 and 2.022(5) to five Cu2) and average Mg-O bond length is 2.092(1) Å (Calvo 1971, 1972). In Cu_3_V_2_O_8_ the Jahn-Teller effect typical of Cu^2+^ (which frequently it is made evident as four short bonds and two longer bonds) distorts the structure, reducing the symmetry from Cmca to P12_1_/c1. The octahedra in Mg_3_V_2_O_8_ are relatively undistorted while in Cu_3_V_2_O_8_ the octahedra are greatly distorted. The CuO_6_ groups sharing an edge have the axially elongated Cu-O bonds nearly parallel. These elongated bond lengths run parallel to a+2c rather than the b axis in Cu_3_V_2_O_8_. In this manner the oxygen which is bonded to three Cu(II) ions and the V(V) ion, can reduced its bond strength whereas in the Mg_3_V_2_O_8_ structure a comparable weakening of the bonds to this over-bonded oxygen atom cannot occur (Calvo 1972).

Cu_2_V_2_O_7_ and Mg_2_V_2_O_7_ structures

Most of divalent metal divanadates, M_2_V_2_O_7_, are polymorphic. Copper divanadate, Cu_2_V_2_O_7_, presents orthorhombic (Calvo 1975), monoclinic (Mercurio-Lavaud and Bernard 1973) and triclinic crystalline forms and Mg_2_V_2_O_7_ presents monoclinic and triclinic symmetries (Calvo 1974).

In orthorhombic α-Cu_2_V_2_O_7_ structure, the V_2_O_7_^4-^ anion contains a crystallographic twofold axis through the bridging oxygen atom with V-O bond lengths ranging from 1.648(5) to 1.743(2) Å and a V-O-V angle of 147.8(5)^o^. In orthorhombic
α-Cu_2_V_2_O_7_ with space group Fdd2 there are only a different crystallographic Cu(II) ion (Cu1), only one crystallographic V(V) ion and four different crystallographic oxygen atoms (O1, O2, O3, O4). Cu1, V1, O2, O3, O4 ions are in 16b general sites and O1 ions are in 8a special sites. Cu(II) ions are bonded strongly to four oxygen atoms with Cu-O bond lengths ranging from 1.880(5) to 1.972(5) Å and weakly to a fifth at 2.542(4) Å [4]. The Cu ion shows fivefold coordination to oxygen atoms with the sixth oxygen atom over 3 Å from the Cu. The shortest Cu-O bond is trans to the empty sixth site in the coordination sphere about Cu.

In monoclinic β-Cu_2_V_2_O_7_ with space group C12/c1 there are also only a different crystallographic Cu(II) ion (Cu1), only one crystallographic V(V) ion and four different crystallographic oxygen atoms (O1, O2, O3, O4). Cu1, V1, O2, O3, O4 ions are in 8f general sites and O1 ions are in 4e special sites. Its structure is characterized by anionic groups (V_2_O_7_)^4-^ clearly individualized and evenly distributed to form layers parallel to the plane x0y. Cu(II) cations are situated in pairs among those thus ensuring the cohesion of the whole structure (Mercurio-Lavaud and Bernard 1973).

The relationship between the α and β-Cu_2_V_2_O_7_ structures is an interesting one. Each structure contains sheets of V_2_O_7_ groups with a staggered configuration and lying on a crystallographic twofold axis. Adjacent anions in the sheet are generated by C centring. The anions in a given sheet are generated by C centring. The anions in a given sheet in α-Cu_2_V_2_O_7_ must be rotated by 25^o^, about the twofold axes, towards the plane of the sheet (the bc plane). Those in the next adjacent sheet must be inverted about the V-V vector before the rotation in order to generate the anion packing of β-Cu_2_V_2_O_7_. After such changes the structure will have a monoclinic cell defined by b, c and 1/2(a+b), in terms of the unit cell of α-Cu_2_V_2_O_7_ (a= 20.645(20), b= 8.383(7),
c= 6.442(12) Å), whose dimensions (a’= 8.411(7), b´=6.448(1), c`=11.14(2) Å and β=112.1(2)^o^) are roughly as in β-Cu_2_V_2_O_7_. Finally, adjacent sheets must slip alternately by about +c/8 and –c/8 in order to accommodate the changed bonding environment of the Cu ions. Each Cu ion must break and reform at least three Cu-O bonds through the transition. These changes seem large for a reversible transformation [4]. All anions with V-O-V bond angles of less than 148ᵒ have strong interactions between the divalent metal ion and the bridging oxygen atom.

In monoclinic Mg_2_V_2_O_7_ with space group P12_1_/c1 there are two different crystallographic Mg(II) ion (Mg1 and Mg2), two crystallographic V(V) ion (V1 and V2) and seven different crystallographic oxygen atoms (O1, O2, O3, O4, O5, O6 and O7). All atoms are in 4e general sites. Mg1 and Mg2 are surrounded by sis oxygen atoms and V(V) is tetrahedrally coordinated. In this crystalline form, one side of the anion has a long, but significant, interaction with a fifth oxygen atom (V-O = 2.440 (4) Å).

The triclinic structure of Mg_2_V_2_O_7_ with PῙ space group consists of chains of V_2_O_7_ groups formed from what, to a first approximation, can be described as VO_4_ tetrahedra. These V_2_O_7_ groups are linked by edge-sharing across centres of symmetry and involve a weak fifth V-O bond (2.869(3) Å) at one end and a somewhat stronger fifth V-O bond at the other end [6]. There are two different crystallographic Mg(II) ions (Mg1, Mg2), two different crystallographic V(V) ions (V1, V2) and seven different crystallographic oxygen atoms (O1, O2, O3, O4, O5, O6, O7). All ions and atoms are in 2i general sites in this structure. Mg(II) ions are all in octahedral coordination.
