# Supplementary figures and images for: Structural characterization and colour of MgxCu3-xV2O8 (0 ≤ x ≤ 3) and MgyCu2-yV2O7 (0 ≤ y ≤ 2) compositions
Source: Springerplus. 2015 Apr 3;4:163. doi: 10.1186/s40064-015-0908-8 (PMC4414859; doi:10.1186/s40064-015-0908-8)

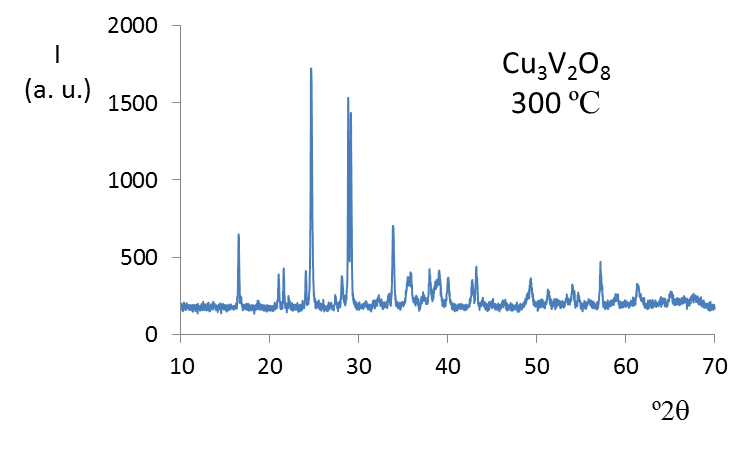

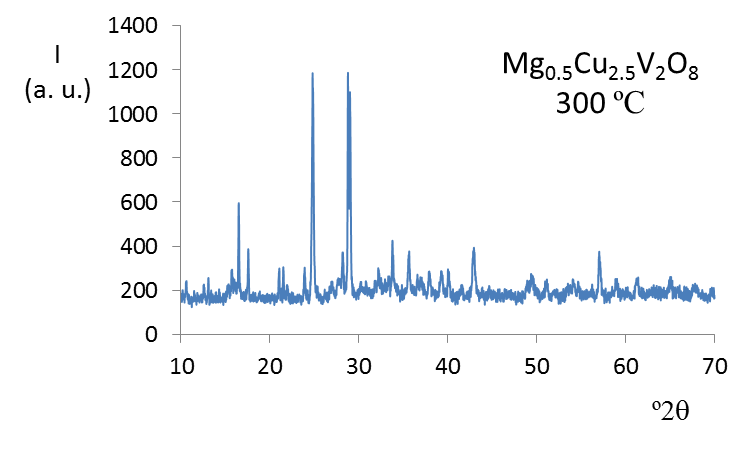

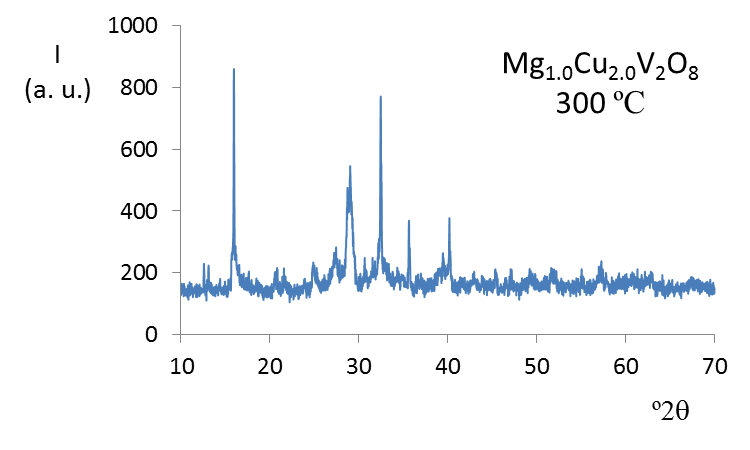


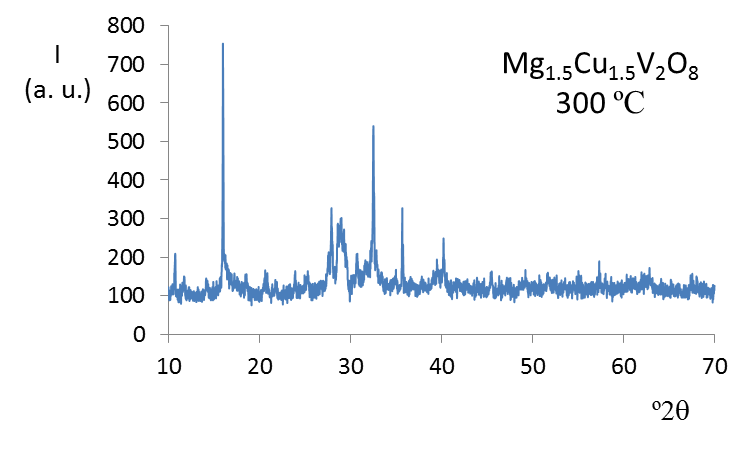

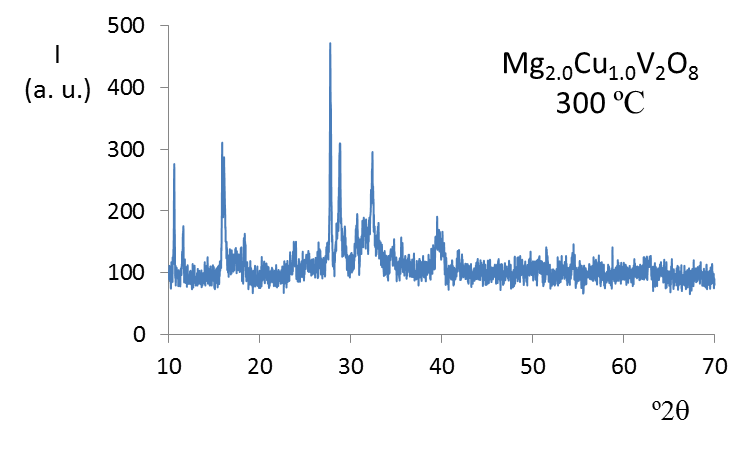

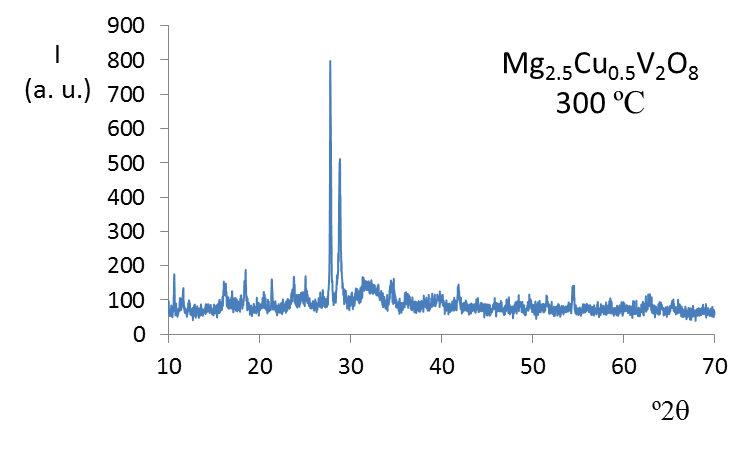

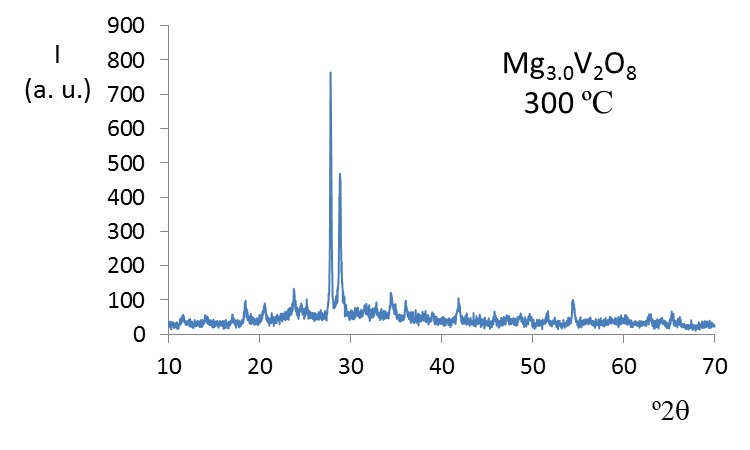

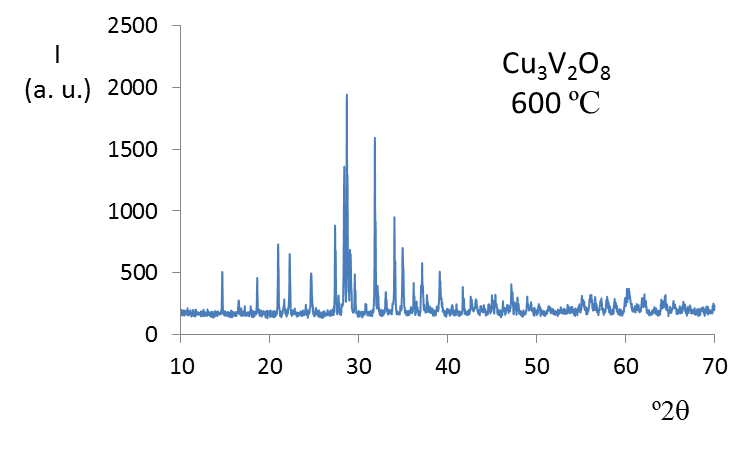

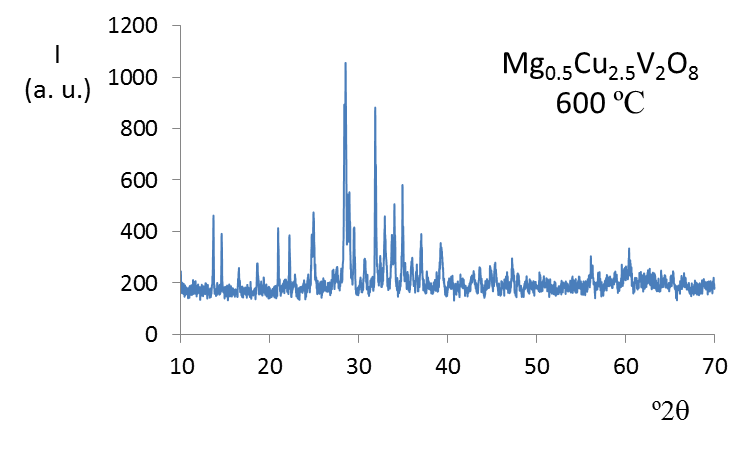

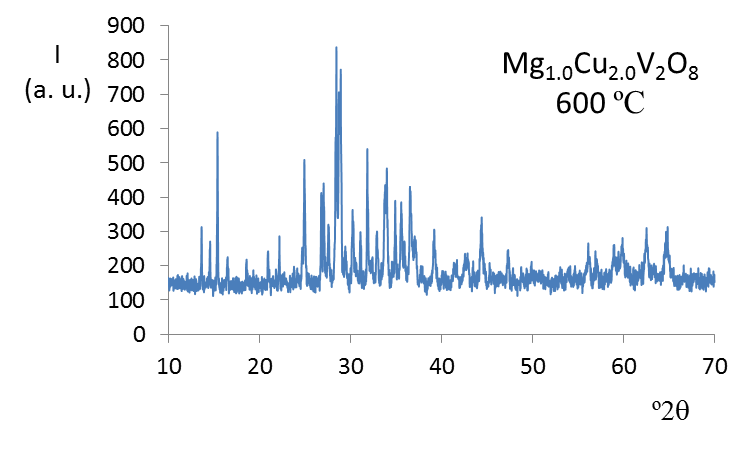

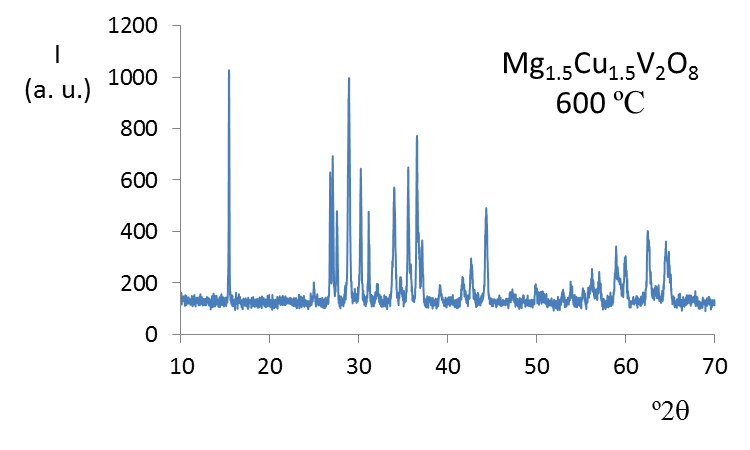

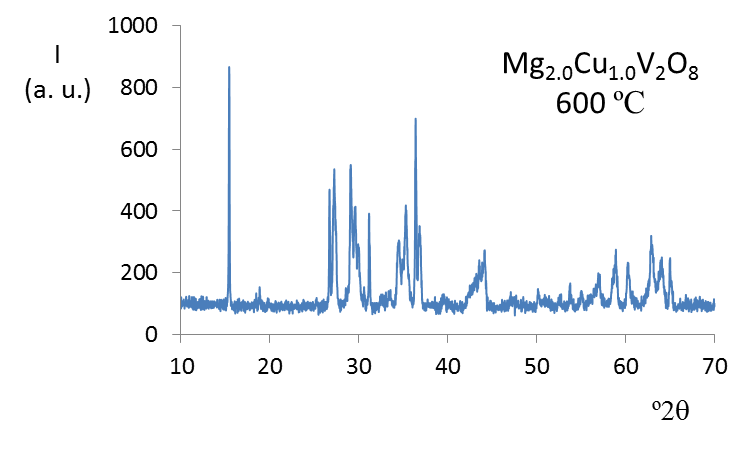

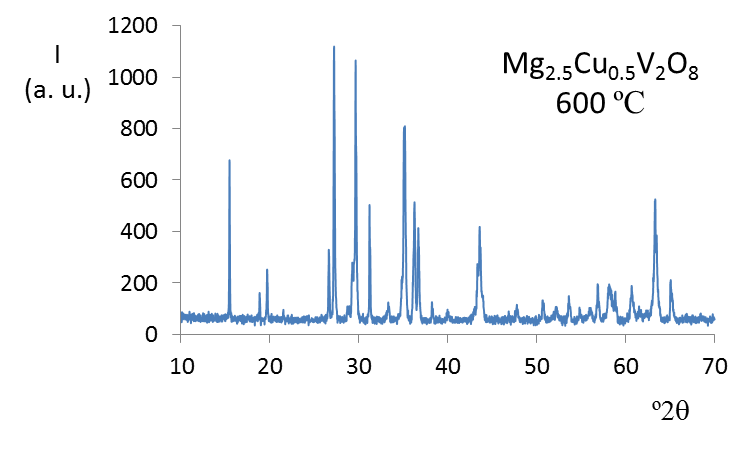

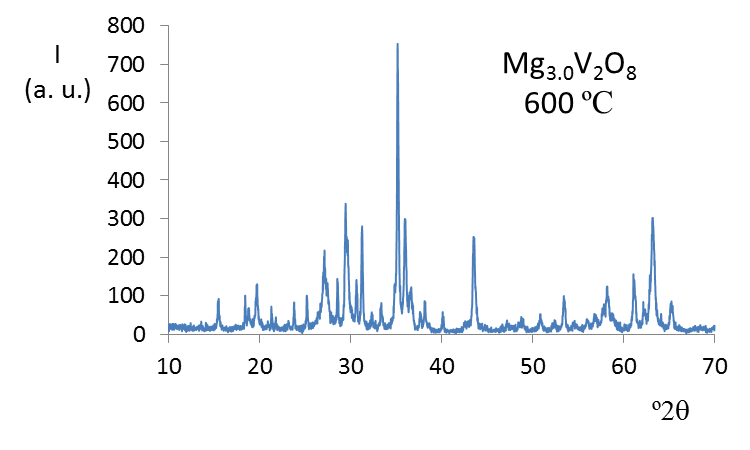

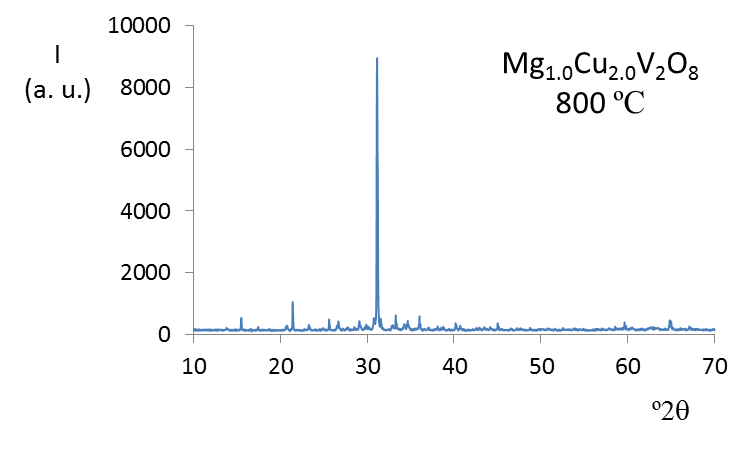

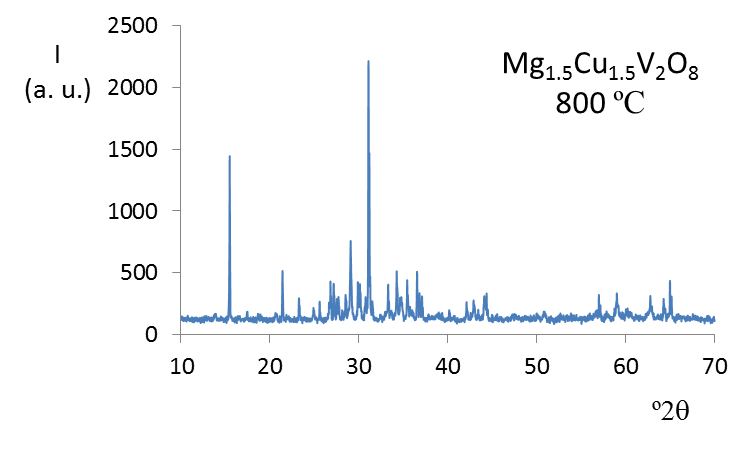

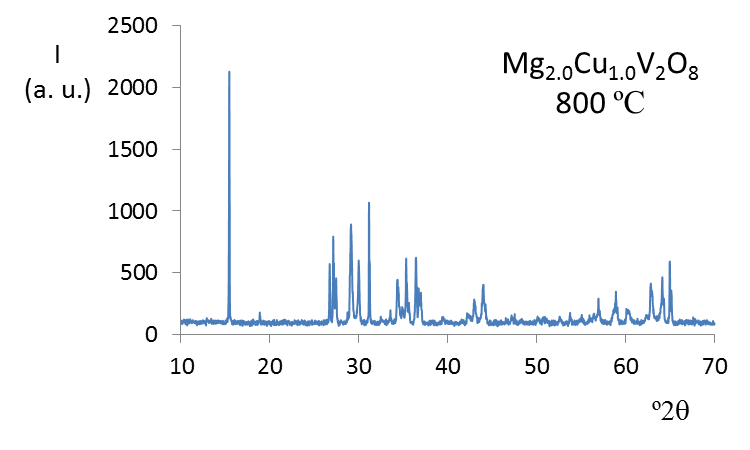

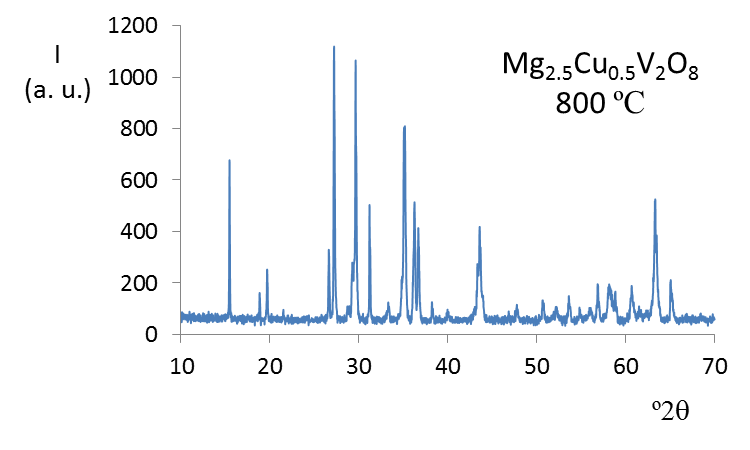

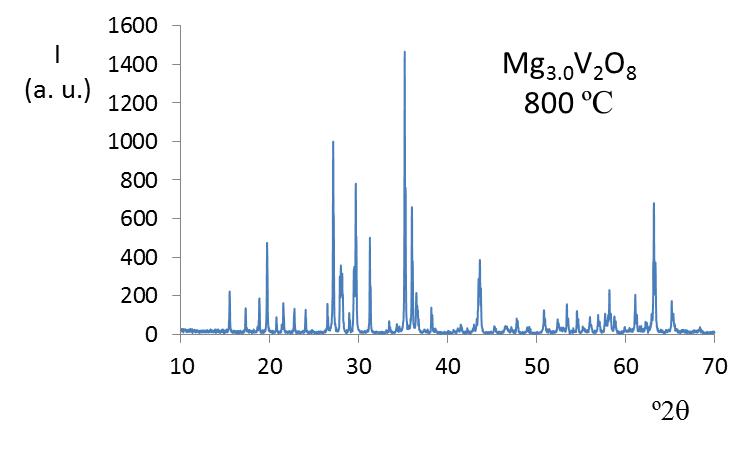

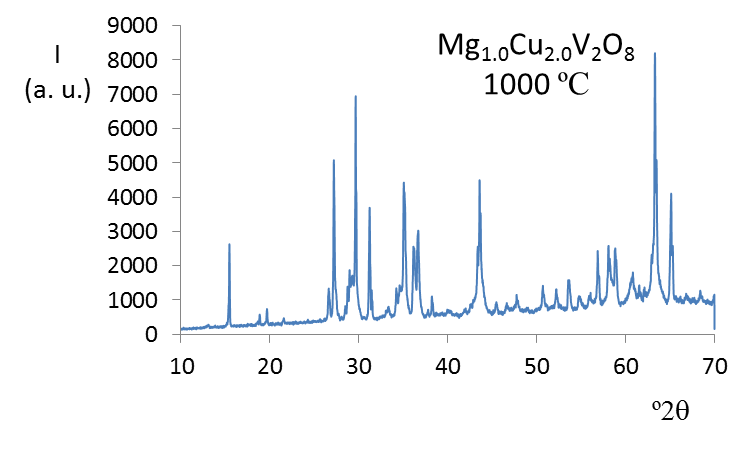

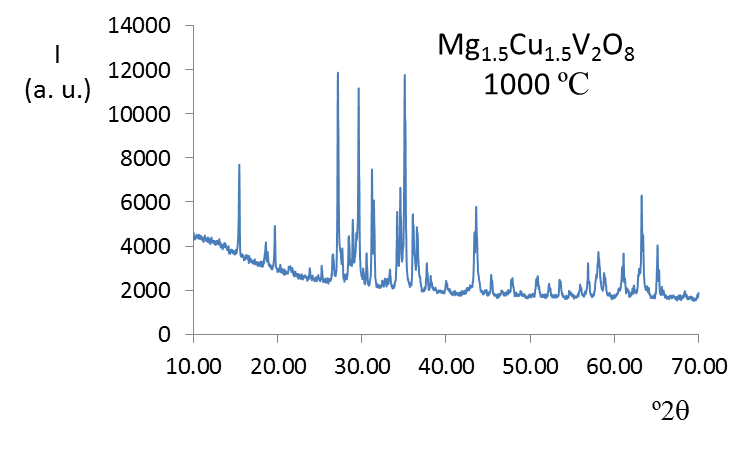

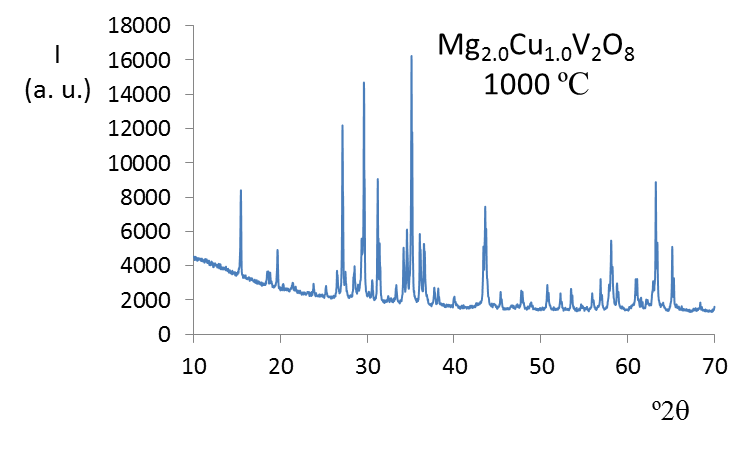

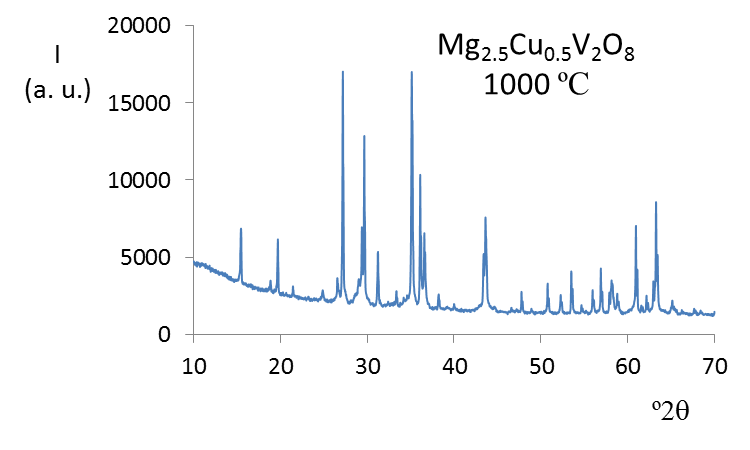


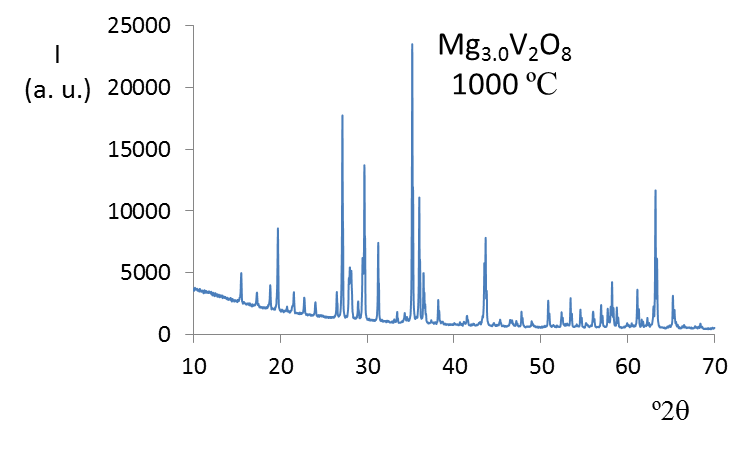

Supplement: Additional file 2: — XRD patterns from Mg x Cu 3-x V 2 O 8 compositions. [file 40064_2015_908_MOESM2_ESM.docx]

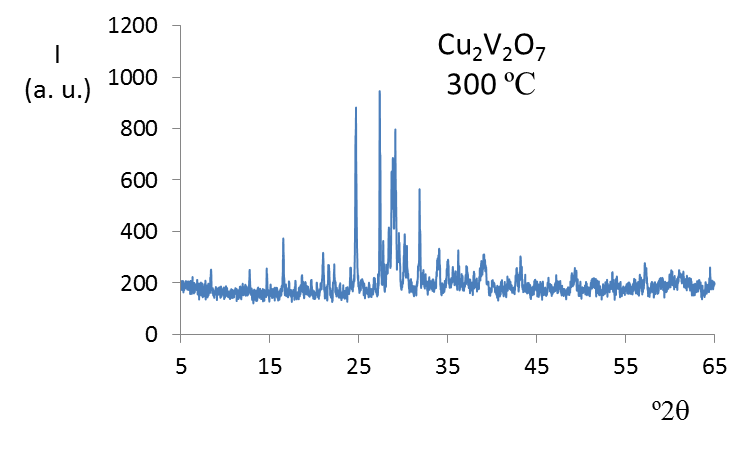

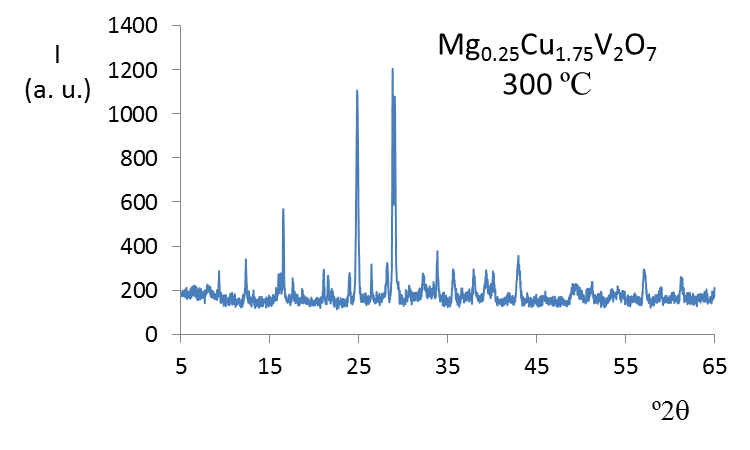

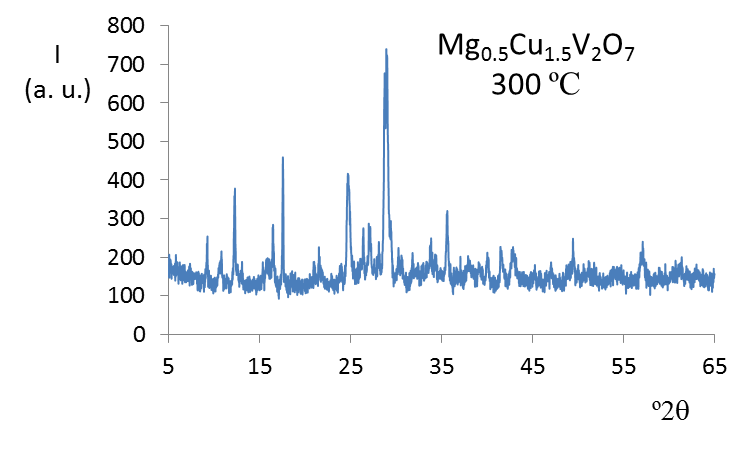

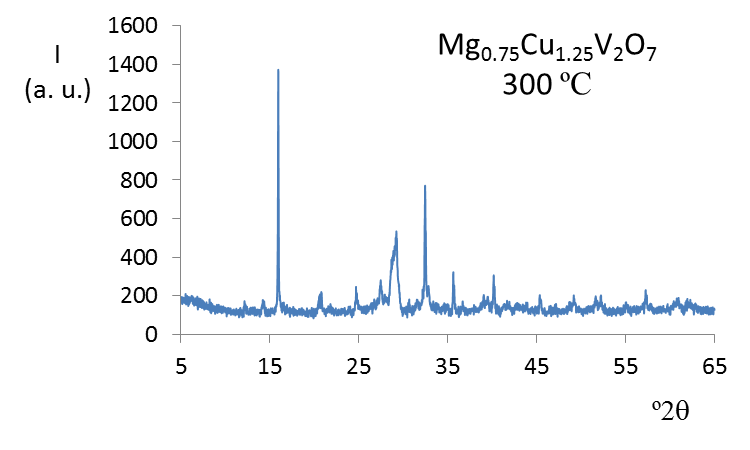

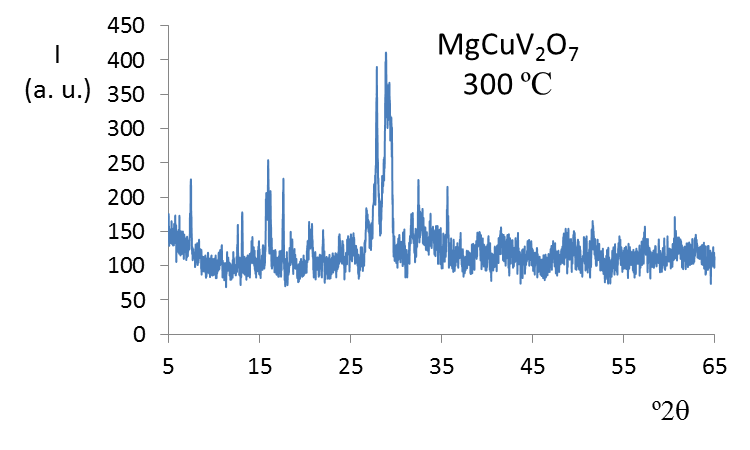

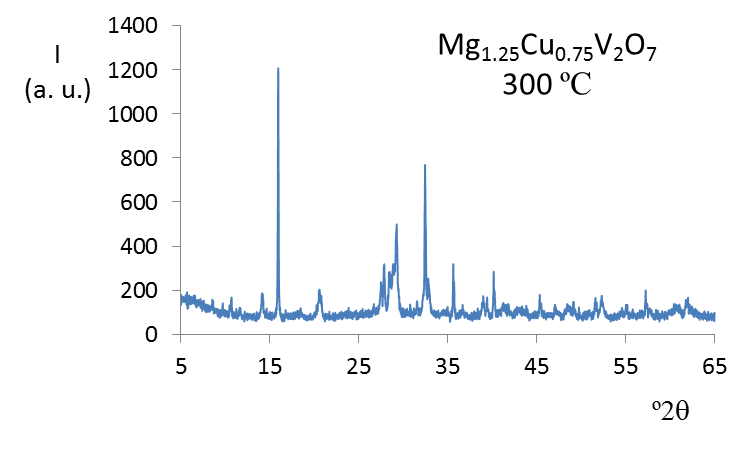

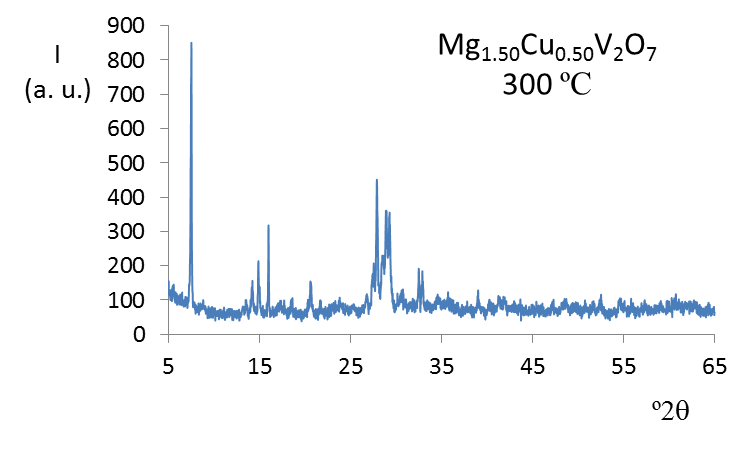

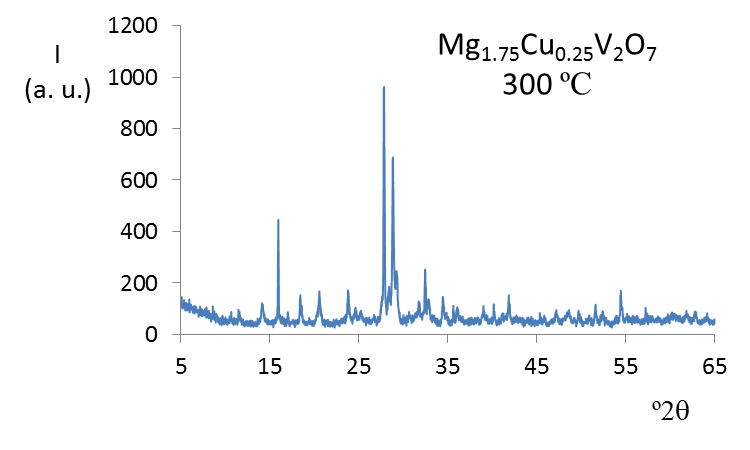

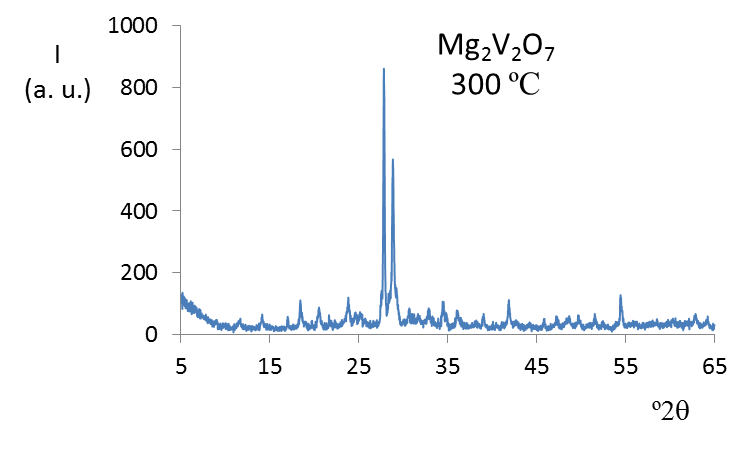

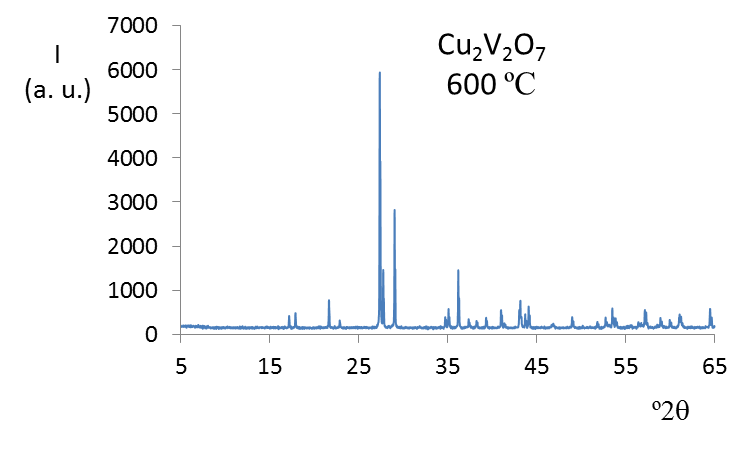

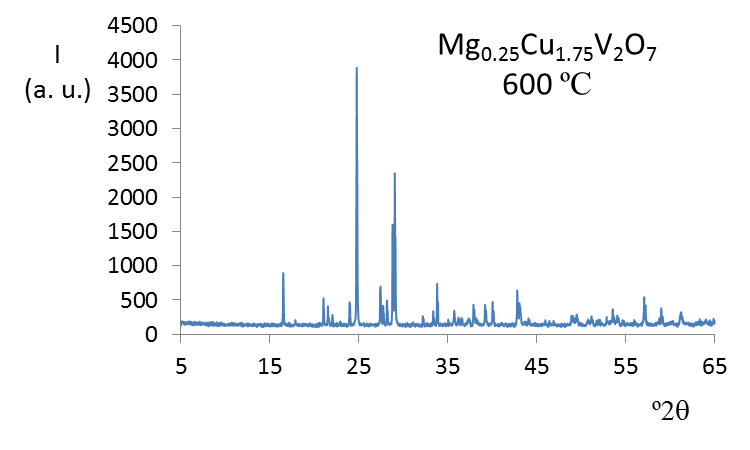

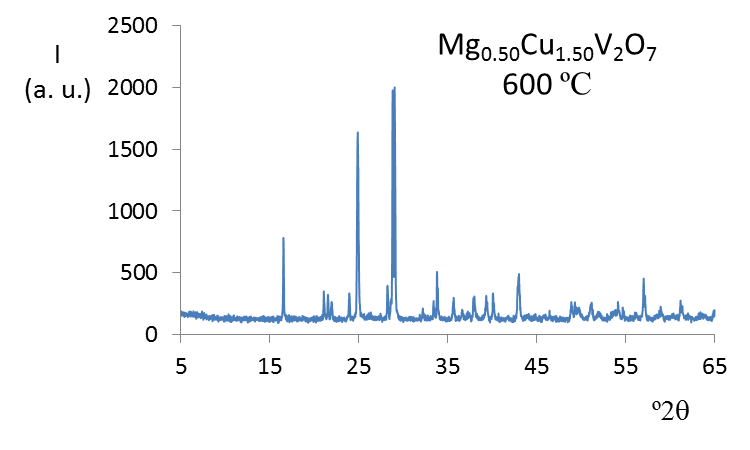

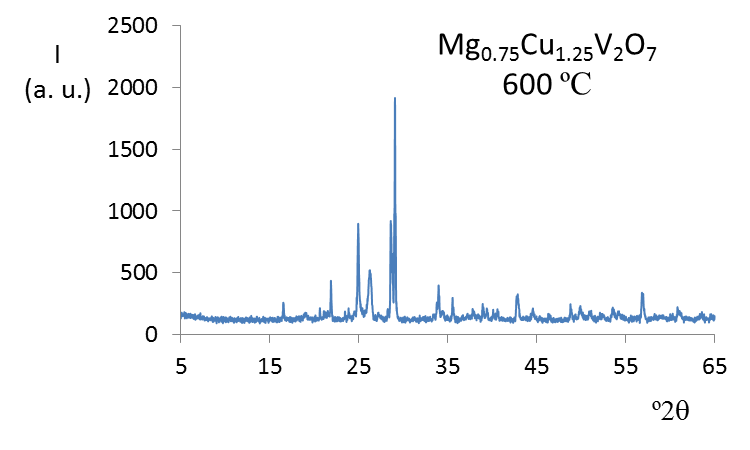

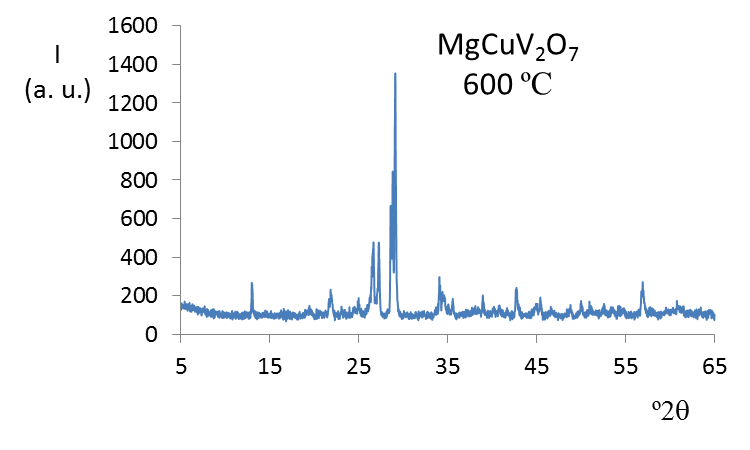

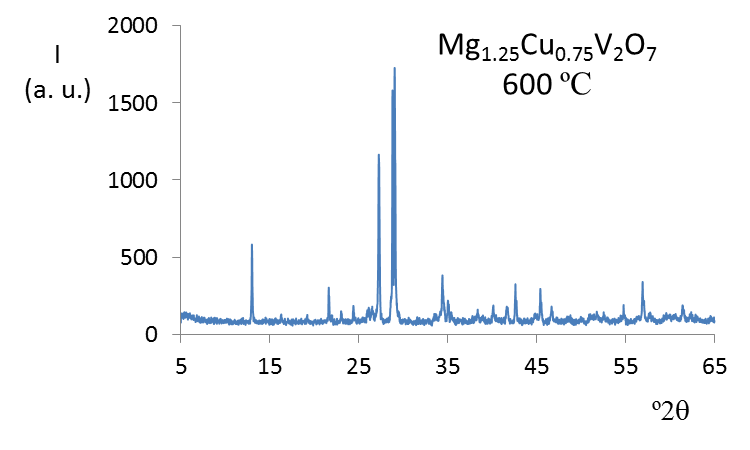

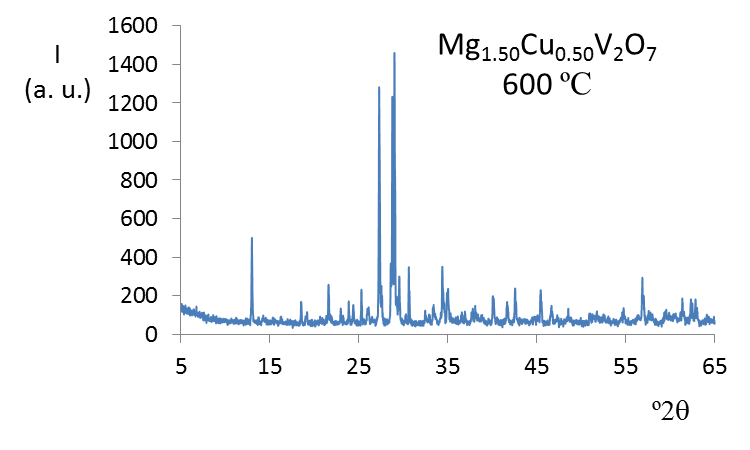

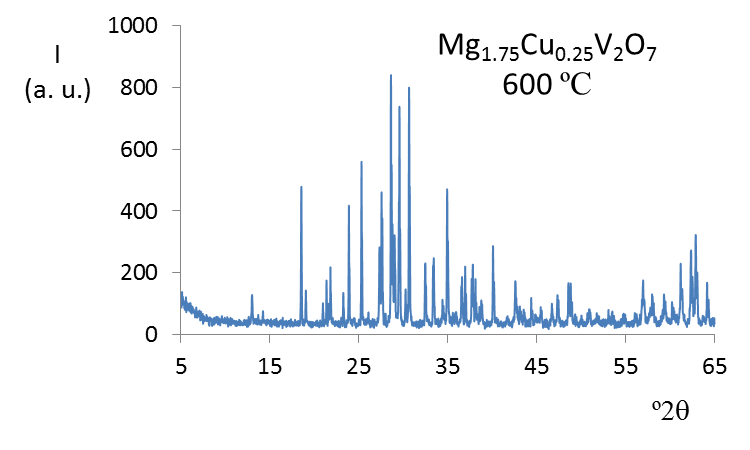

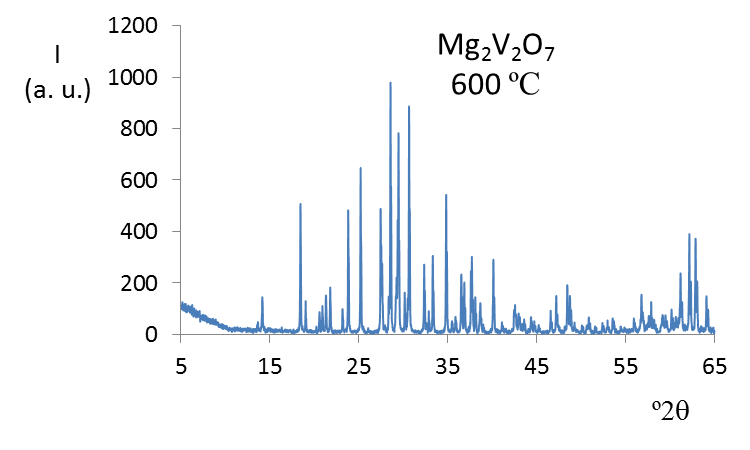


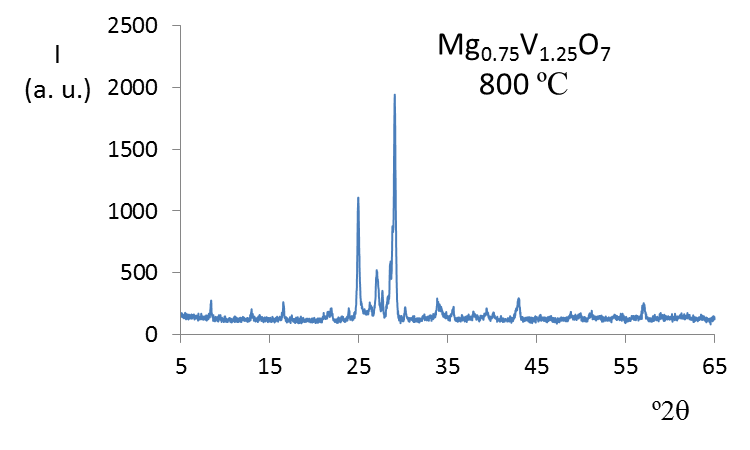

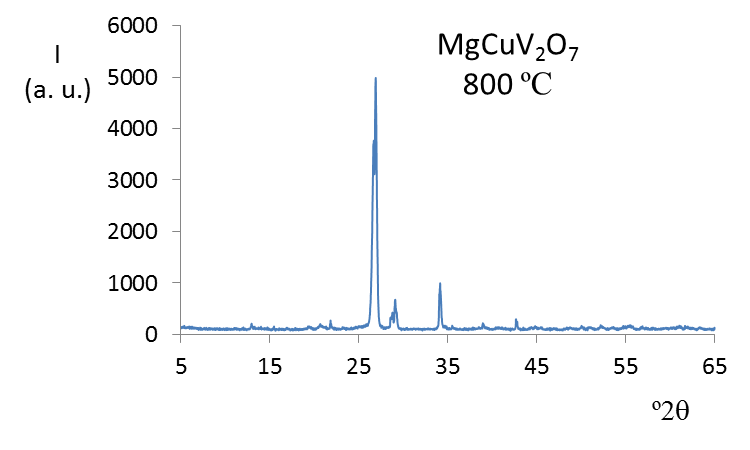

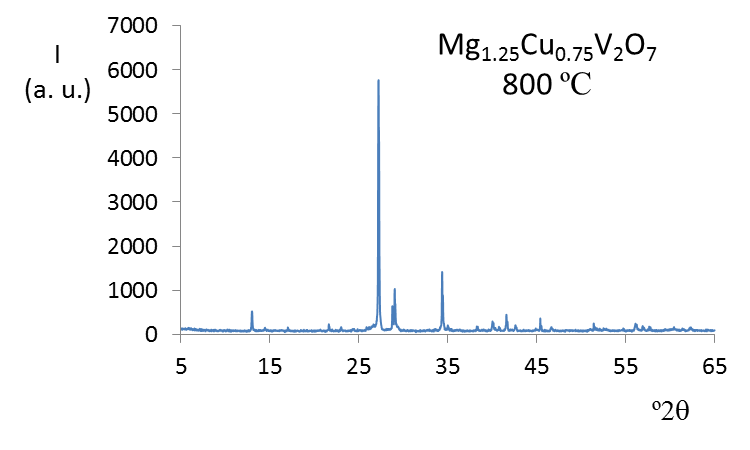

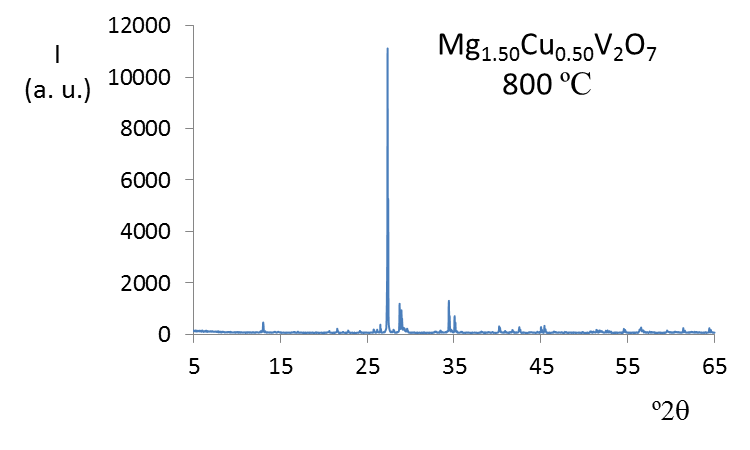

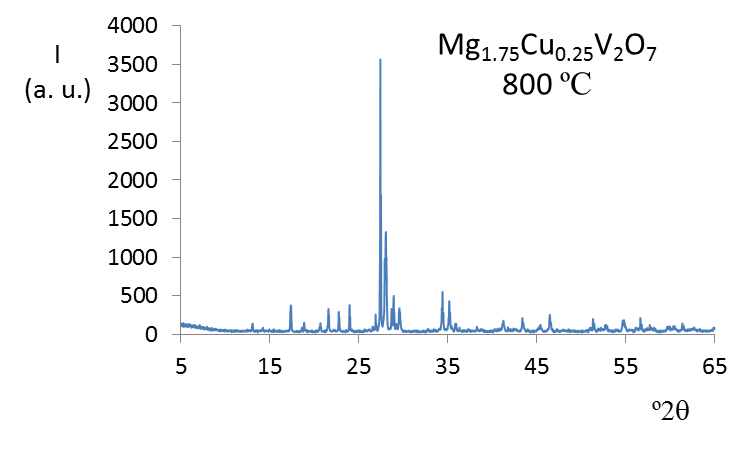

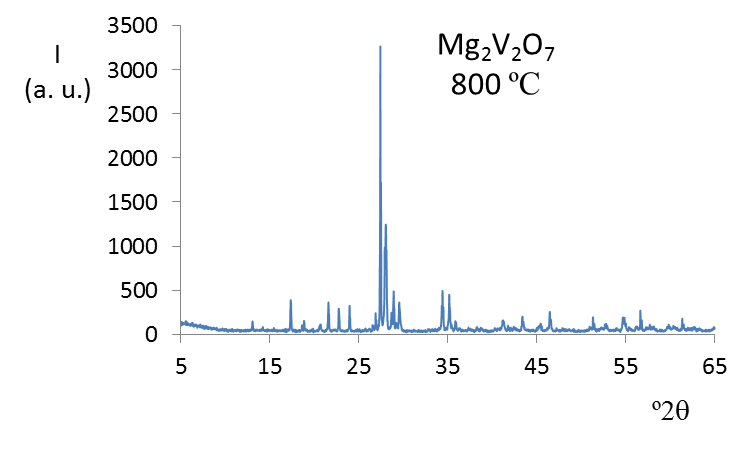

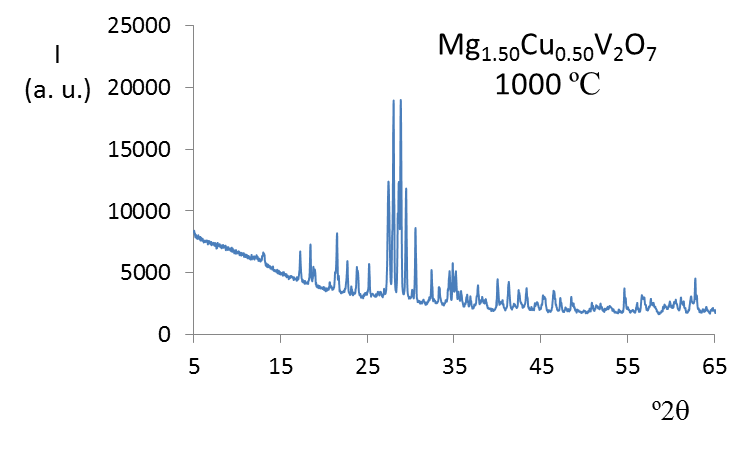

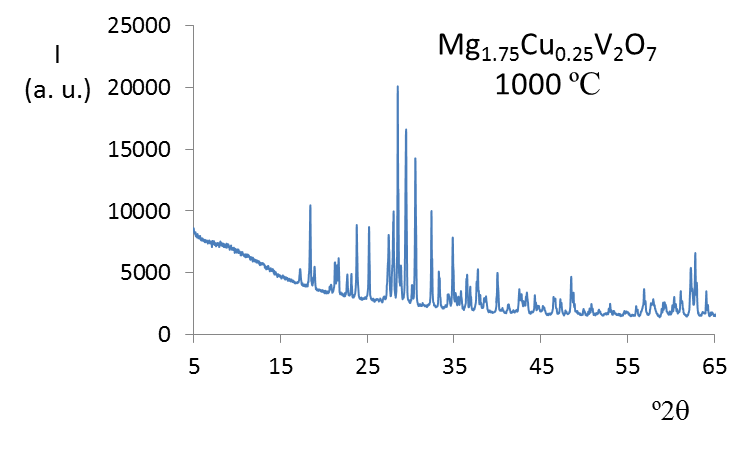

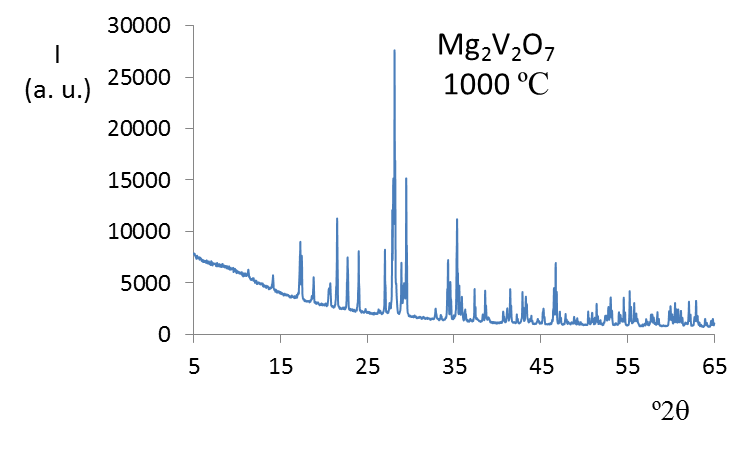

Supplement: Additional file 3: — XRD patterns from Mg y Cu 2-y V 2 O 7 compositions. [file 40064_2015_908_MOESM3_ESM.docx]
